# How effective are remote and/or digital interventions as part of alcohol and drug treatment and recovery support? A systematic review and meta‐analysis

**DOI:** 10.1111/add.70021

**Published:** 2025-03-24

**Authors:** Irene Kwan, Helen Elizabeth Denise Burchett, Wendy Macdowall, Preethy D'Souza, Claire Stansfield, Dylan Kneale, Katy Sutcliffe

**Affiliations:** ^1^ Evidence for Policy & Practice information (EPPI) Centre, Social Research Institute University College London London UK; ^2^ Faculty of Public Health and Policy London School of Hygiene and Tropical Medicine London UK

**Keywords:** alcohol use disorder, digital interventions, drug use disorder, meta‐analysis, remote interventions, systematic review

## Abstract

**Background and Aims:**

Although remote drug/alcohol interventions have been widely reviewed, their effectiveness specifically for people in treatment remains unclear. We aimed to systematically review the effectiveness of remote interventions (delivered by telephone or computer) in alcohol/drug treatment and recovery support.

**Methods:**

We searched 29 databases including Medline and PsycINFO for randomised controlled trials (RCTs) of remote interventions for adults diagnosed with alcohol/drug use disorder conducted in Organization for Economic Co‐operation and Development (OECD) countries published 2004–2023. We grouped interventions according to whether they supplemented or replaced/partially replaced in‐person care. We used random effects meta‐analyses to estimate pooled odds ratios (OR) for relapse, and standardised mean differences (SMD) for days of alcohol/drug use. We appraised outcomes using Cochrane Risk of Bias 2.

**Results:**

We identified 34 RCTs (6461 participants) evaluating 42 remote interventions, with diverse therapeutic approaches. Over 70% of outcomes were judged to be at high risk‐of‐bias. When remote interventions supplemented in‐person care, there was a 39% lower odds of relapse [17 interventions; OR 0.61; 95% confidence interval (CI) = 0.46, 0.81; *P* = 0.001; I^2^ = 40.3%) and a reduction in the mean days of use (17 interventions; SMD −0.18; 95% CI = −0.28 to −0.08; *P* = 0.001; I^2^ = 27.3%) compared with in‐person care alone. When remote interventions replaced/partially replaced in‐person care, there was a 49% lower odds of relapse (7 interventions; OR 0.51; 95% CI = 0.34, 0.76; *P* = 0.001; I^2^ = 39.7%) and a very slight and uncertain reduction in mean days of use (8 interventions; SMD −0.08; 95% CI = −0.24 to 0.07; *P* = 0.301; I^2^ = 48.4%) compared with in‐person care. Subgroup analyses by type of substance and therapeutic approach were mixed and inconclusive.

**Conclusions:**

Remote interventions which supplement in‐person alcohol/drug treatment appear to reduce relapse and days of use. The evidence is less conclusive regarding remote interventions that replace/partially replace in‐person care due to a smaller body of evidence and uncertainty (days of use). High risk‐of‐bias means findings should be interpreted with caution.

## INTRODUCTION

It is estimated that over 2% of the world's population has either an alcohol or illicit drug use disorder [1, 2, 3]. Globally, deaths because of drug use have risen sharply over the past decade [3, 4] and substance misuse is an important contributor to global disease burden [5, 6]. Further, both alcohol and drug misuse are associated with an array of adverse social consequences including violence, accidents, relationship problems, barriers to employment and crime [7, 8].

Alcohol/drug interventions comprise those aimed at prevention (targeting the general population), early intervention (targeting those who may be at risk of substance use disorder), and treatment and recovery support (for people with diagnosed substance use disorders) [6, 9, 10]. Structured alcohol/drug treatment typically consists of a comprehensive package of specialist interventions, which includes an assessment of needs and a clear recovery care plan, with goal setting and regular reviews. The package may contain pharmacological interventions, psychosocial interventions and other services as appropriate. ‘Recovery support’ covers a range of interventions delivered alongside or after structured treatment that are designed to reinforce treatment gains [9]. In this article, we consider ‘treatment’ interventions as encompassing treatment and/or recovery support.

The availability of interventions that can be delivered remotely and/or digitally (e.g. by telephone, smartphone, internet or computer) has grown in recent years. During the coronavirus disease 2019 (COVID‐19) pandemic, remote interventions were deployed across health sectors to provide support and treatment to patients during lockdowns [11]. The adoption of these technologies has continued post‐COVID [12, 13]. There is evidence that digital health interventions can have favourable effects in terms of both costs and health outcomes across a range of health conditions [14, 15].

There are potential disadvantages and advantages to using remote interventions within drug and alcohol treatment. It is possible that faced with the challenges of time constraints, lack of training and inadequate resources, services may use remote interventions for cost‐saving purposes rather than to optimise patient outcomes [16]. However, they have been found to help overcome some key challenges to seeking treatment, such as stigma [17], and increase access to services especially for rural populations [18]. Further, evidence suggests that remote interventions can foster engagement with treatment and encourage self‐management [19, 20].

**FIGURE 1:**
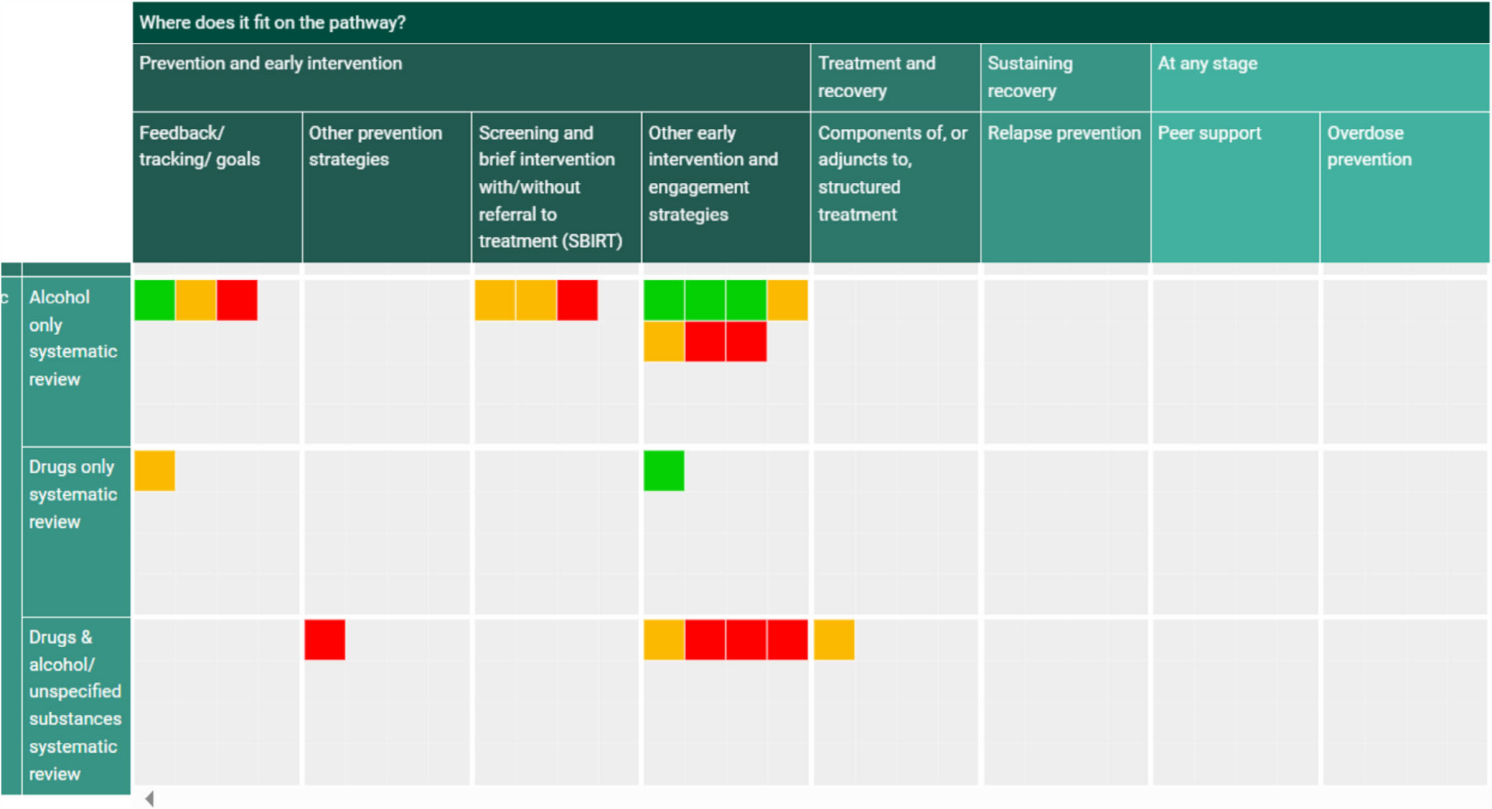
Map of systematic reviews on digital alcohol and drug interventions. *Note*: The map indicates 21 reviews were available; this is because three of the 18 reviews each contain two relevant analyses.

Before the meta‐analyses reported here, we published a comprehensive map of the evidence on digital drug/alcohol interventions for prevention, early intervention, treatment and recovery [10]. Although our initial searches for the map identified over 50 reviews, the majority were excluded either because they combined a mix of prevention and treatment interventions and did not distinguish between them in their analyses or because they did not include a synthesis of findings. Our map included 18 systematic reviews, but only one synthesized evidence specifically on treatment interventions (Figure 1). This moderate quality review found a decrease in drug use for digital community reinforcement and contingency management (CRCM) [*n* = 6 studies; *n* = participants not stated; pooled effect size (g) = 0.39; 95% CI = 0.26, 0.52; *P* ≤ 0.001] [21]. Given that CRCM is only one type of treatment intervention that can be delivered digitally, there remains a gap in terms of review‐level evidence on the range of digital treatment interventions.

The current study aimed to fill a gap identified in our original map (i.e. to synthesise the evidence on the effectiveness of the full range of digital interventions for alcohol/drug treatment). However, the increase in use of telephone interventions during the COVID‐19 pandemic highlighted the need to also include delivery mode. Therefore, for the purpose of this review, we define remote interventions as therapeutic interventions delivered either via landline telephone, on fixed computers (location‐specific), on‐line via the internet or through a mobile application on smartphones and/or text messages. These interventions may be provided either as a supplement to in‐person treatment or as a replacement/partial replacement for in‐person treatment components. They involve tailored interaction to support people to build commitment, motivation and belief in their capacity to reduce or stop their alcohol/drug consumption and to develop a range of cognitive and behavioural skills, to reach their personal goals.

Our research questions were:

- Does supplementing in‐person treatment with remote intervention(s) enhance its effectiveness?
- When provided as a replacement or partial replacement to in‐person treatment, are remote interventions no less effective?
- Does the effect of remote interventions vary according to the target substance (alcohol, drugs, alcohol and drugs)?
- Does the effect of remote interventions vary according to the type of therapeutic approach (remote recovery support, remote talking therapy and self‐guided therapy)?

The work is part of a wider systematic review, which also used qualitative comparative analysis (QCA) to explore the critical features associated with ‘most’ and ‘least’ effective interventions, which is reported elsewhere [22].

## METHODS

In this article, we report four meta‐analyses of randomised studies and four sub‐group analyses. The protocol for the wider review was prospectively registered with PROSPERO [23]. We followed the Preferred Reporting Items for Systematic reviews and Meta‐Analyses (PRISMA) guidelines [24] (see Appendix S1 and S2).

### Inclusion criteria

Table 1 presents the inclusion criteria for this review and meta‐analysis.

**TABLE 1:** Inclusion criteria.

	To be included, studies had to:
Participants	Focus on adults ≥18 years of age receiving treatment for drug and/or alcohol dependence
Intervention	Evaluate interventions that are delivered remotely (i.e. on‐line, by telephone or through a mobile smartphone app), parallel to or as a component of in‐person alcohol/drug treatment or recovery support, are interactive and which support users to build commitment, motivation and develop a range of skills for reducing alcohol/drug use.
Comparisons	Have any type of comparator including in‐person treatment, no intervention, or an alternative remotely delivered intervention.
Study designs	Be RCTs
Outcomes	Report either relapse or days of alcohol/drug use outcomes.
Geographical location	Be conducted in OECD countries
Date	Be published in or after 2004
Language	Be written in the English language
Publication types	Be reported in published journal articles only; no protocols, posters or conference abstracts

Abbreviation: OECD, Organization for Economic Co‐operation and Development.

### Information sources and search strategy

We identified literature from an earlier evidence map [10] and updated and expanded searches to meet the scope of this review until August 2023. Searches on 29 bibliographic databases and other on‐line resources were undertaken during November and December 2021, as specified in the protocol [23]. These were re‐run on 14 July 2023 in Medline (OVID), PscyINFO (OVID), Social Sciences Citation Index [WoS (Web of Science)] and Emerging Sources Citation Index (WoS) as these had yielded all the included studies. OpenAlex was used for forward, backward and citation‐relations searches (5 July 2023) and for recommender searches (OpenAlex records added between 4 June 2023 and 20 August 2023 only). The searches were conducted by our information scientist (C.S.) in collaboration with H.B. and K.S. using the concepts of (1) telephone, video, digital or remote support; (2) intervention, treatment, or service context; and (3) drug use or alcohol misuse. The MEDLINE strategy is included in Appendix S3.

### Study selection and data extraction

After de‐duplication, we imported the search results into the systematic review software EPPI‐Reviewer [25] and used its machine learning function to iteratively prioritise records during the screening and automatically remove records likely to be of low relevance. Screening ceased once a plateau of included records was observed and was supported by other indicators (e.g. comparison with an estimated baseline inclusion rate for the 2021 searches and applying machine learning classifiers to rank and scan some of the remaining unscreened records for the 2023 searches) [25, 26]. Details of the priority screening process are available in Appendix S4. At least two reviewers (from K.S., H.B., W.M., P.D. and I.K.) independently screened a sample of records of titles/abstracts, as well as full reports from potentially eligible citations, against the inclusion criteria. Disagreements were resolved through consensus. Once agreement rates were adequate (>90%), both titles/abstracts and full reports were screened by a single reviewer. We extracted key descriptive information from included studies using a bespoke coding tool, covering study design, intervention characteristics, population, context and outcomes (see Appendix S5). We piloted the tools to ensure consistency and clarity. Two reviewers extracted data independently (from K.S., H.B., W.M., P.D. and I.K.) and differences were resolved by discussion or consulting a third reviewer within the review team.

### Study categorisation

Studies were categorised by: outcome measured, type of therapeutic approach, substance targeted and by type of evaluation.

#### Outcome measured

Our two outcomes were relapse (abstinence not maintained) and days of alcohol/drug use. To ensure comparability, we extracted outcome data for the most commonly reported timepoint, which was at the end of intervention. Outcomes were measured by self‐report and/or toxicology.

#### Type of therapeutic approach

We categorised remote interventions into three main types; any interventions not of these types were categorised as ‘other’:

- Remote recovery support: a range of interventions to check up on recovery progress, maintain or improve motivation, support recovery goals, identify risks of or actual relapse and/or facilitate access to treatment or other recovery services if required.
- Remote talking therapy: remote, synchronous group or individual counselling sessions, typically based on cognitive, behavioural, psychological, psychodynamic or 12‐step interventions and delivered by a trained, qualified therapist or counsellor.
- Self‐guided therapy: a structured programme containing different activities that people work through themselves, such as self‐monitoring and skill development.

#### Type of substance use targeted

We categorised substance use into three main types:

- Alcohol only.
- Mixed drugs and alcohol.
- Drugs only.

#### Type of evaluation

Studies broadly fell into two evaluation categories: (1) those which evaluated remote interventions as a supplement to in‐person treatment, compared to in‐person care alone and (2) those that evaluated remote interventions as a replacement or partial replacement to in‐person treatment, compared to exclusively in‐person treatment. Partial replacement interventions were those where some of the in‐person components were replaced with remote components. Replacement interventions were those where, following assessment and diagnosis, intervention participants received only remote intervention.

We created this categorisation because we assumed that for interventions delivered as a supplement to in‐person treatment, to demonstrate success, outcomes should be superior to the comparator. However, for interventions replacing or partially replacing in‐person treatment, we assumed that to demonstrate success, outcomes should not be unacceptably worse than the comparator. It should be noted that most of the individual studies were not formally designed or conducted as inferiority/superiority analyses [27].

### Analyses

We conducted four meta‐analyses, covering each outcome (relapse/days of use) for each type of evaluation (supplement/replacement) (see Table 2). Within the models, some studies provided multiple effect sizes for different intervention arms. Where arms were compared to a common control, adjustments were made to the effective sample size of the control group in line with common, pragmatic meta‐analytic practice, to (partially) address unit of analysis issues [28].

**TABLE 2:** Details of the four meta‐analyses.

Research questions	Outcome	Meta‐analysis
Does supplementing in‐person treatment with remote intervention(s) enhance its effectiveness?	Relapse	**1**
	Days of use	2
When provided as a replacement or partial replacement to in‐person treatment components, are remote interventions no less effective?	Relapse	3
	Days of use	4

### Assessing risk‐of‐bias

We applied the Cochrane risk‐of‐bias (RoB) 2 tool [29] to assess RoB at the outcome level, assigning assessments of ‘low risk’, ‘some concerns’ or ‘high risk’ to determine each outcome's performance across five domains: (1) RoB arising from the randomisation process; (2) RoB because of deviations from the intended interventions; (3) RoB because of missing data; (4) RoB in measurement of outcomes; and (5) RoB in selection of the reported results. The judgments within each domain led to an overall RoB judgment of ‘low risk’, ‘some concerns’ or ‘high risk’ for the outcome being assessed. At least two reviewers (from K.S., H.B., W.M., P.D. and I.K.) rated each study's outcome(s) and discordant ratings were resolved by discussion or consulting a third team member. We report the RoB assessments for individual outcomes. Additionally, to ensure that the review findings are interpreted with appropriate caution we report the summary of the RoB across studies with each meta‐analysis finding and in reporting the overall conclusions [30].

### Statistical analysis

We analysed data based on the intention‐to‐treat (ITT) or modified ITT samples. When appropriate, five reviewers (D.K., K.S., H.B., W.M. and P.D.) transformed outcome data to harmonise odds ratio (OR) and standardised mean difference (SMD) into the same metric: ‘abstinence’ data into ‘relapse’ and ‘days of alcohol/drug non‐use’ to ‘days of use’. We performed random‐effects meta‐analyses using STATA software [31] to produce pooled summary estimates of effect sizes, presented in forest plots. We analysed relapse and days of use by calculating the OR and SMD, respectively, with 95% CI. We assessed heterogeneity of effect sizes using the I^2^ statistic in which heterogeneity was deemed to be substantial if I^2^ was greater than 50%. We also conducted subgroup analyses to examine whether effects varied according to the type of substance targeted by the intervention (alcohol, mixed drugs and alcohol or drugs only) and the type of therapeutic approach used (remote recovery support, remote talking therapy and self‐guided therapy). Publication bias was assessed using Egger's test and visual inspection of funnel plots [32].

## RESULTS

### Search results

**FIGURE 2:**
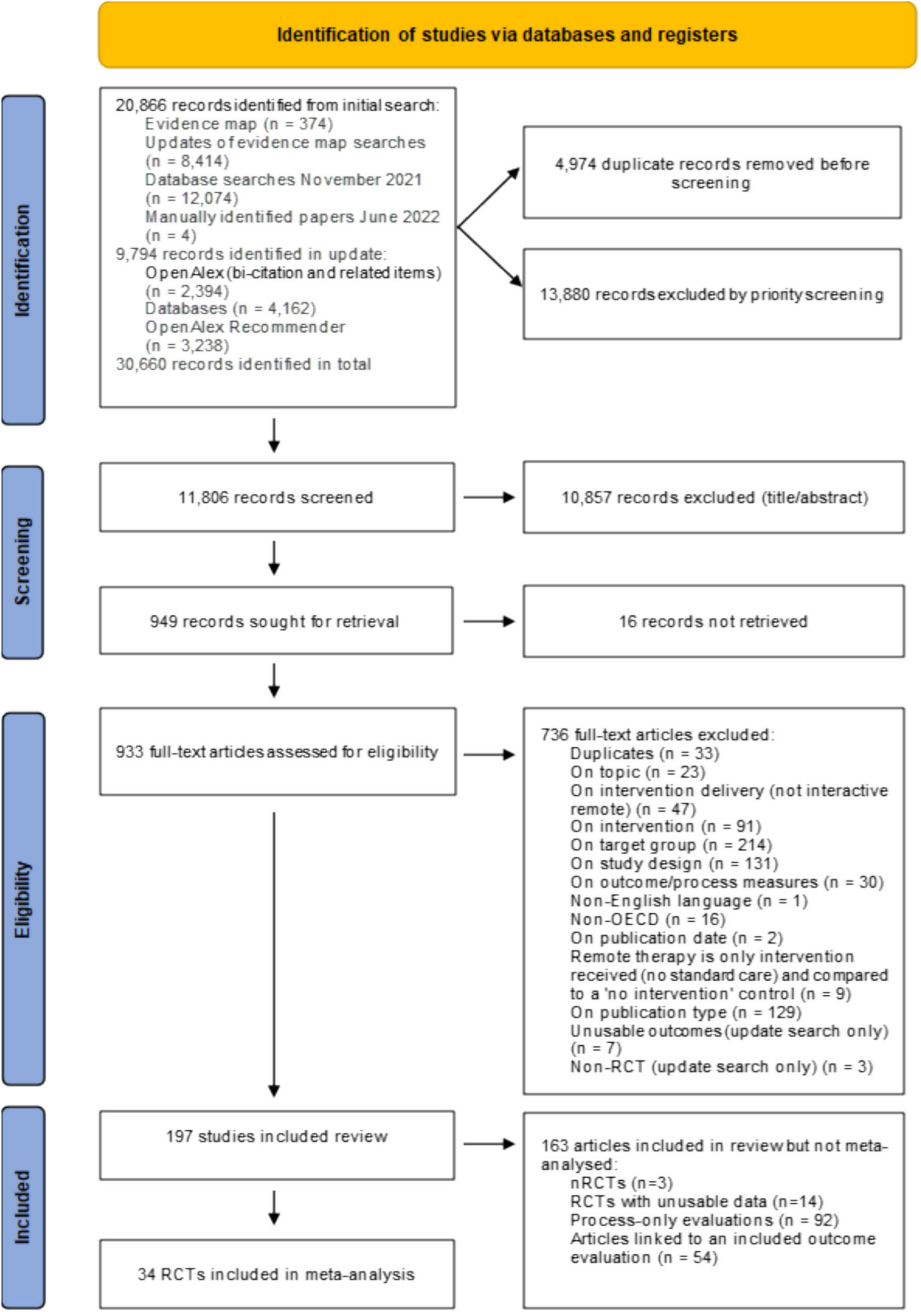
Preferred Reporting Items for Systematic Reviews and Meta‐Analyses (PRISMA) flow diagram of studies through the review.

Systematic searches in the wider review identified 25 686 records after removing duplicates [22]. Priority screening resulted in the exclusion of 13 880 records likely to be of low relevance (see Appendix S4 for details of the priority screening process). The remainder were screened manually on title/abstract and 949 warranted full‐text screening. Of these 949 records, 34 RCTs met the criteria for inclusion in the meta‐analyses (see Figure 2).

### Study characteristics

#### Description of studies

The majority of the 34 RCTs (*n* = 27) were published after 2010, and most (*n* = 26) were conducted in the United States. Table 3 presents a summary of the characteristics of the included studies. Of the 34 RCTs included in the meta‐analyses, six were multi‐arm trials involving two (*n* = 4) or three remote interventions (*n* = 2), resulting in a total of 42 remote interventions. Meta‐analyses 1 and 2, focusing on remote interventions as a supplement to in‐person treatment, each contain 17 interventions. Meta‐analyses 3 and 4, focusing on remote interventions as a replacement or partial replacement are smaller, containing seven and eight interventions, respectively.

**TABLE 3:** Summary of participants and intervention characteristics (*n* = 34 RCTs).

Interventions	Study ID (reference) country	Participant characteristics	Intervention type	Outcome
			Intervention(s) (I)	Risk‐of‐bias
			Control (C)	
			Intervention duration / timing of outcome measurement used in meta‐analysis	
			Substance	
Interventions offered as a supplement to in‐person treatment (meta‐analyses 1 and 2) (*n* = 30)				
1.	Brooks 2010 [41] USA	*N* = 26 Mean age ~ 43 years > 90% African American 50% female	Self‐guided therapy I: TAU + online Therapeutic education system (TES) + cash incentives (*n* = 14) C: TAU + cash incentives at random (*n* = 12) 8 weeks / not reported Drugs (cocaine)	Days of useRoB2: High
2.	Carroll 2008 [42] USA	*N* = 77 Mean age = 42 years 64% ethnic minorities 43% female	Self‐guided therapy I: TAU + CBT4CBT (*n* = 39) C: TAU (*n* = 38) 8 weeks / not reported Mixed substances	Days of useRoB2: high
3.	Carroll 2014 [43] USA	*N* = 101 Mean age = 42 years 40% ethnic minorities 60% female	Self‐guided therapy I: TAU + CBT4CBT (*n* = 47) C: TAU (*n* = 54) 8 weeks / not reported Mixed substances	RelapseRoB2: some concerns Days of useRoB2: some concerns
4.	Christensen 2014 [44] USA	*N* = 170 Mean age = 34 years 5% ethnic minorities 46% female	Self‐guided therapy I: TAU + online community reinforcement (*n* = 92) C: TAU (*n* = 78) 12 weeks / not reported Drugs (opioids)	Days of useRoB2: some concerns
5.	DeMartini 2018 [36] USA	Liver transplant candidates *N* = 15 Mean age = 51 years 7% ethnic minorities 27% female	Remote recovery support I: TAU + text messages (*n* = 8) C: TAU (*n* = 7) 8 weeks / not reported Alcohol	RelapseRoB2: some concerns
6.	Fals‐Stewart 2010 [45] USA	*N* = 160 Mean age = 33 years 48% ethnic minorities 32% female	Self‐guided therapy I: TAU + computer‐assisted cognitive rehabilitation (*n* = 80) C: TAU + computer‐assisted typing tutorial (*n* = 80) 8 weeks / 9 weeks Mixed substances	Days of useRoB2: high
7.	Farabee 2013 [46] USA	*N* = 302 Mean age = 37 years 44% ethnic minorities 27% female	Remote talking therapy I: TAU + telephone based continuing care (*n* = 249) C: TAU (*n* = 53) 12 weeks / 39 weeks Drugs (stimulants)	RelapseRoB2: high Days of useRoB2: high
8.	Farren 2014 [47] Ireland	*N* = 55 Mean age = 45 years NR % ethnic minorities 28% female	Self‐guided therapy I: TAU + computerised CBT (31) C: TAU + placebo (cognitive stimulating intervention)(*n* = 24) 4 weeks / 12 weeks Alcohol	Days of useRoB2: high
9.	Farren 2022 [48] Ireland	*N* = 111 Mean age ~45 years NR % ethnic minorities 48% female	Self‐guided therapy I: TAU + UControlDrink app (*n* = 54) C: TAU (*n* = 57) 26 weeks / not reported Alcohol	Days of useRoB2: high
10.	Godley 2010 [49] USA	*N* = 104 Mean age = 32 years 24% ethnic minorities 40% female	Remote recovery support I: TAU + telephone support (*n* = 51) C: TAU (*n* = 53) 12 weeks / 12 weeks Mixed substances	Days of useRoB2: high
11‐13.	Graser 2021 [50] Switzerland	*N* = 240 Mean age = 50 years NR % ethnic minorities 31% female	Remote talking therapy (I1 and I2); remote recovery support (I3) I1: TAU + telephone calls (high frequency) (*n* = 51) I2: TAU + telephone calls (low frequency) (*n* = 64) I3: TAU + text messages (*n* = 61) C: TAU (*n* = 64 – nb 3‐arm trial so control split into 3 groups of 21.33 for analysis) 26 weeks / not reported Alcohol	RelapseRoB2: high
14.	Gustafson 2014 [51] USA	*N* = 349 Mean age = 38 years 20% ethnic minorities ~40% female	Remote recovery support I: TAU + ACHESS app (Addiction – Comprehensive Health Enhancement Support System) (*n* = 170) C: TAU (*n* = 179) 34 weeks / 16 weeks Alcohol	RelapseRoB2: high
15.	Hyland 2023 [52] Sweden	*N* = 264 Mean age = 51 years NR % ethnic minorities 66% female	Self‐guided therapy I: TAU + internet‐based CBT (*n* = 132) C: TAU (*n* = 132) 12 weeks / 52 weeks Alcohol	Days of useRoB2: high
16.	Kelpin 2022 [35] USA	*N* = 63 Mean age = 41 years 87% ethnic minorities 100% female	Self‐guided therapy I: TAU+ CBT4CBT (*n* = 34) C: TAU (*n* = 29) ~4 weeks / 4 weeks Mixed substances	RelapseRoB2: some concerns Days of useRoB2: some concerns
17.	Kiluk 2016^a^ [53] USA	*N* = 68 Mean age = 43years 66% ethnic minorities 35% female	Self‐guided therapy I1: TAU + CBT4CBT (*n* = 22) C: TAU (*n* = 22 – nb. 2 arm trial – other arm in replacement / partial replacement table below – so control split into 2 groups of 11 for analysis) 8 weeks / 8 weeks Alcohol	Days of useRoB2: some concerns
18.	Lucht 2020 [54] Germany	*N* = 462 Mean age = 45 years NR % ethnic minorities 23% female	Remote recovery support I: TAU + text messages (*n* = 230) C: TAU (*n* = 233) 52 weeks / not reported Alcohol	RelapseRoB2: some concerns Days of useRoB2: some concerns
19‐20.	McKay 2010 [55] USA	*N* = 252 Mean age = 43 years 89% ethnic minorities 36% female	Remote recovery support I1: TAU + telephone monitoring (*n* = 83) I2: TAU+ telephone monitoring + counselling (*n* = 83) C: TAU (*n* = 86 – nb. 2 arm trial so control split into 2 groups of 43 for analysis) 78 weeks / not reported Alcohol	RelapseRoB2: some concerns
21‐22.	McKay 2013 [56] USA	*N* = 321 Mean age = 43years 93% ethnic minorities 24% female	Remote talking therapy I1: TAU + telephone monitoring + counselling (*n* = 106) I2: intervention 1 + incentives (*n* = 107 C: TAU (*n* = 108 – nb. 2 arm trial so control split into 2 groups of 54 for analysis) 104 weeks / not reported Mixed substances	RelapseRoB2: high
23‐24.	Mundt 2006 [57] USA	*N* = 60 Mean age = 42 years 5% ethnic minorities 45% female	Self‐guided therapy I1: TAU + Daily IVR reporting + follow‐up (*n* = 20) I2: TAU + Daily IVR reporting without follow‐up (*n* = 20) C: TAU (*n* = 20 – nb. 2 arm trial so control split into 2 groups of 10 for analysis) 26 weeks / not reported Alcohol	RelapseRoB2: high
25.	Paris 2018 [40] USA	*N* = 92 Mean age = 43 years 100% ethnic minorities 33% female	Self‐guided therapy I: TAU + CBT4CBT (*n* = 43) C: TAU (*n* = 49) 8 weeks / 8 weeks Mixed substances	Days of useRoB2: some concerns
26.	Taştekin 2022 [34] Turkey	*N* = 53 Mean age = 30 years NR % ethnic minorities 0% female	Self‐guided therapy I: TAU + computer‐assisted cognitive remediation (*n* = 26) C: TAU (*n* = 27) 4 weeks / 8 weeks Drugs (opioids)	RelapseRoB2: high
27.	Tetrault 2020 [58] USA	*N* = 58 Mean age = 44 years 40% ethnic minorities 43% female	Self‐guided therapy I: TAU + CBT4BCT (*n* = 30) C: TAU (*n* = 28) 8 weeks / not reported Mixed substances	Days of useRoB2: high
28.	Timko 2019a [59] USA	*N* = 298 Mean age = 50 years 24% ethnic minorities 5% female	Remote recovery support I: TAU + enhanced telephone monitoring(*n* = 148) C: TAU (*n* = 150) 12 weeks / 12 weeks	Days of useRoB2: high
29.	Timko 2019b [39] USA	*N* = 406 Mean age = 45 years 27% ethnic minorities 8% female	Remote recovery support I: TAU + telephone monitoring (*n* = 207) C: TAU (*n* = 199) 12 weeks / 12 weeks Mixed substances	Days of useRoB2: high
30.	Verduin 2013 [33] USA	War veterans *N* = 41 Mean age = 51 years 34% ethnic minorities 0% female	Computer simulation game I: TAU + alcohol relapse prevention game (*n* = 22) C: TAU + educational slide show (*n* = 19) 12 weeks / 4 weeks	RelapseRoB2: high
Remote intervention as a replacement or partial replacement for in‐person treatment (meta analyses 3 and 4) (*n* = 12)				
1.	Campbell 2014 [60] USA	*N* = 507 Mean age = 35 years 44% ethnic minorities 38% female	Self‐guided therapy I: TAU + TES + contingency management (*n* = 255) C: TAU (*n* = 252) 12 weeks / not reported Mixed substances	RelapseRoB2: low Days of useRoB2: low
2.	Chaple 2016 [37] USA	Prison inmates *N* = 494 Mean age = 37 years 51% ethnic minorities 30% female	Self‐guided therapy I: E‐TES (*n* = 249) C: TAU (*n* = 245) 12 weeks / variable (12 weeks after prison release) Mixed substances	Days of useRoB2: high
3.	Gonzales 2014 [61] USA	*N* = 81 Mean age = 20 years 57% ethnic minorities 27% female	Remote recovery support I: mobile‐phone based aftercare: Project ESQYIR (Educating and Supporting inQuisitive Youth In Recovery) (*n* = 40) C: TAU (*n* = 41) 12 weeks / 12 weeks Mixed substances	RelapseRoB2: High
4.	Johansson 2021 [62] Sweden	*N* = 301 Mean age = 50 years NR % ethnic minorities 39% female	Self‐guided therapy I: internet‐based CBT (*n* = 151) C: in‐person CBT (*n* = 150) 12 weeks / 12 weeks Alcohol	Days of useRoB2: high
5.	Kiluk 2016^a^ [53] USA	*N* = 68 Mean age = 43 years 66% ethnic minorities 35% female	Self‐guided therapy I2: CBT + monitoring (*n* = 24) C: TAU (*n* = 22 – nb. 2 arm trial—see other arm in supplement table above—so control split into two groups of 11 for analysis) 8 weeks / 8 weeks Alcohol	Days of useRoB2: some concerns
6.	Kiluk 2018 [63] USA	*N* = 137 Mean age = 36 years 66% ethnic minorities 25% female	Self‐guided therapy I: CBT4CBT + monitoring (*n* = 38) C^b^: TAU (*n* = 50) 12 weeks / 12 weeks Alcohol	RelapseRoB2: high Days of useRoB2: high
7.	McKay 2004 [64] USA	*N* = 359 Mean age = 42 years 77% ethnic minorities 18% female	Remote talking therapy I: Telephone monitoring + brief counselling (*n* = 102) C^b^: TAU (*n* = 122) 12 weeks / not reported Mixed substances	RelapseRoB2: high Days of useRoB2: high
8‐10	McKay 2022 [65] USA	*N* = 262 Mean age = 47 years 18% ethnic minorities 29% female	Remote talking therapy (I1); remote recovery support (I2), remote talking therapy + remote recovery support (I3) I1: TAU + telephone monitoring + counselling (*n* = 59) I2: TAU + ACHESS on mobile phone app (*n* = 68) I3: intervention 1 + intervention 2 (*n* = 70) C: TAU (*n* = 65 – nb. 3 arm trial so control spit into three groups of 21.66 for analysis) 52 weeks / 26 weeks Alcohol	RelapseRoB2: high
11	Tiburcio 2018 [66] Mexico	*N* = 74 Mean age = ~24 years NR % ethnic minorities 12% female	Self‐guided therapy I: Web‐based Help Program for Drug Abuse and Depression C^b^: TAU (*n* = 26) 8 weeks / 4 weeks Mixed substances	Days of useRoB2: high
12	Wolitzky‐Taylor 2018 [38] USA	Participants had comorbid mental health problems *N* = 97 Mean age = 36 years 28% ethnic minorities 43% female	Remote talking therapy I: TAU + CALM ARC (*n* = 56) C: TAU (*n* = 41) Mixed substances 7 weeks / 26 weeks	Days of useRoB2: high

^a^ This study contained two arms—one supplementing and one replacing in‐person treatment.

^b^ Control with two arms, only TAU included in meta‐analysis.

Abbreviations: ACHESS, addiction – Comprehensive Health Enhancement Support System; CALM ARC, coordinated anxiety learning and management for addiction recovery centre; CBT, cognitive behavioural therapy; IVR, interactive voice response; NR, not reported; RoB2, risk‐of‐bias 2; TAU, treatment as usual; TES, therapeutic education system.

#### Participant characteristics

In total there were 6461 participants in the studies, with sample sizes ranging from 15 to 507. Participants had a mean age between 20 and 51 years, were predominantly male and Caucasian. Thirty‐one studies included both male and female participants; two studies were male only [33, 34] and one was female only [35]. There were single studies focusing on participants with special characteristics: liver transplant candidates [36]; military veterans [33] and prison inmates [37]. Two studies involved people with co‐morbid mental health conditions [38, 39] and one US study included only Spanish‐speaking individuals in the trial of a culturally adapted program [40] (Table 3).

#### Intervention characteristics

Of the 42 interventions, 20 targeted alcohol use only, 17 targeted a mix of substances (which could include alcohol) and five targeted specific drugs (two cocaine, two opioids and one stimulants). Thirty interventions were evaluated as a supplement to in‐person care, and 12 replaced or partially replaced in‐person treatment. The majority of the interventions (24/42) lasted between 4 and 12 weeks (Table 3).

Twenty‐one interventions were self‐guided therapy, ten were remote talking therapy and nine were remote recovery support. One was a computer simulation game, and another combined remote recovery support and remote talking therapy (Table 4).

**TABLE 4:** Types of remote interventions by substance targeted (*n* = 42 interventions).

Types of remote intervention/substance use focus	Remote recovery support (*n* = 10)	Remote talking therapy (*n* = 9)	Self‐guided therapy (*n* = 21)	Other (video game) (*n* = 1)	RRS + RTT^a^ (*n* = 1)	Total
Alcohol	6	4	8	1	1	20
Mixed	4	4	9	0	0	17
Drugs	0	1	4	0	0	5
**Total**	**10**	**9**	**21**	**1**	**1**	**42**

Abbreviations: RRS, recovery support; RTT, remote talking therapy.

^a^ One intervention combined both RRS and RTT.

Interventions were delivered via the internet (*n* = 18), text messages (*n* = 3), mobile/cell phone app (*n* = 4), telephone calls (*n* = 11), interactive voice response (IVR) (*n* = 2) and computer game (*n* = 1). Four interventions involved multi‐modal delivery.

#### Evaluation types

As illustrated in Table 3, all interventions were compared to treatment as usual (TAU). TAU type and intensity varied across studies, but it involved some form of in‐person care, for example in‐person cognitive behaviour therapy, group therapy or 12‐step programmes. Of the 42 interventions, 30 were evaluated as a supplement to TAU and compared to TAU alone. The remainder (*n* = 12) were evaluated as a replacement or partial replacements for in‐person care and compared to TAU. Most of these (*n* = 10) fully replaced in‐person care, of which half (*n* = 5) were delivered to new patients as a ‘stand‐alone’ treatment intervention and half (*n* = 5) were offered to patients who were transitioning from a more intensive phase of care such as in‐patient treatment. The remainder (*n* = 2) were offered to new patients as a partial replacement for in‐person care, for example, to replace 2 of 6 hours of in‐person care.

### RoB evaluation

In total, 49 outcomes from 42 interventions were assessed for RoB. There were 24 assessments on the outcome of ‘relapse’ and 25 assessments on the outcome of ‘days of use’, as seven interventions reported both outcomes.

**FIGURE 3:**
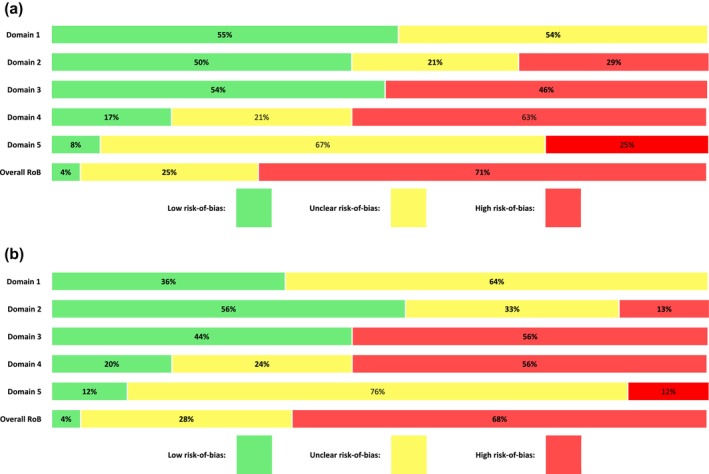
(a) Risk‐of‐bias per domain for outcomes of relapse (*n* = 24 assessments). (b) Risk‐of‐bias per domain for outcomes of days of alcohol/drug use (*n* = 25 assessments).

All the outcomes, except two, were judged to have high RoB (34/49; 69%) or as having some concerns (13/49, 25%) [see Figure 3(a) and Figure 3(b) for summaries and Appendix S6 for each study's RoB]. Only two outcomes (from the same study) were judged to have a low RoB in all the five domains [60]. Across the 49 assessments, the domains most commonly judged to have high RoB were domain 3 (RoB because of missing outcome data) and domain 4 (RoB in measurement of outcome). Bias because of missing data was judged to be high for 46% (11/24) for relapse and 56% (14/25) for days of use outcomes. In domain 4 (RoB because of measurement of outcome), 63% (15/24) of results measuring relapse and 56% (14/25) of results measuring days of use were judged to be at high risk.

### Effectiveness of interventions

Meta‐analysis 1: Do remote intervention(s) evaluated as a supplement to in‐person treatment reduce relapse compared to in‐person treatment alone?

**FIGURE 4:**
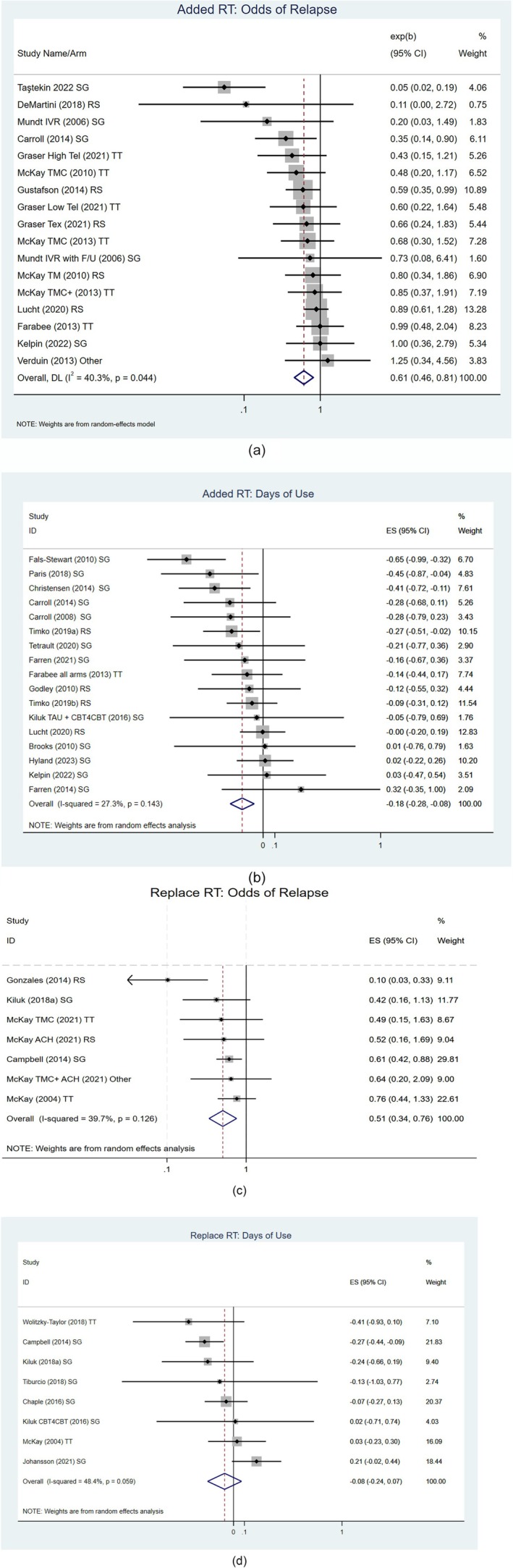
(a) Forest plot of comparison: remote intervention(s) plus in‐person treatment/recovery support versus in‐person treatment alone, outcome: odds of relapse. (b) Forest plot of comparison: remote intervention(s) plus in‐person treatment/recovery support versus in‐person treatment alone, outcome: days of alcohol/drug use. (c) Forest plot of comparison: remote intervention(s) as a replacement or partial replacement for in‐person treatment versus in‐person treatment alone, outcome: odds of relapse. (d) Forest plot of comparison: remote intervention(s) as replacement or partial replacement for in‐person treatment versus in‐person treatment alone, outcome: days of alcohol/drug use.

The odds of relapse was 39% lower among people receiving a remote intervention plus in‐person treatment, compared to those who received in‐person treatment alone (OR = 0.61; 95% CI = 0.46, 0.81; *P* = 0.001; 17 interventions; 2104 participants; I^2^ = 40.3%) [see Figure 4(a)]. There was significant heterogeneity and RoB was judged to be ‘high’ or of ‘some concerns’ across all outcomes. In addition, there was some evidence from Egger's test for publication bias that smaller studies with larger impacts in reducing the odds of relapse were more likely to be published (*P* = 0.015). The funnel plot (not shown here) also suggested that this may be the case.

Meta‐analysis 2: Do remote intervention(s) evaluated as a supplement to in‐person treatment reduce days of alcohol/drug use compared to in‐person treatment alone?

There was a reduction in days of alcohol/drug use among people receiving remote intervention plus in‐person treatment compared to those who received in‐person treatment alone (SMD = −0.18; 95% CI = −0.28 to −0.08; *P* = 0.001; 17 interventions; 2566 participants; I^2^ = 27.3%) [see Figure 4(b)]. RoB was judged to be ‘high’ or of ‘some concerns’ across all outcomes. For this set of studies, there was no evidence of publication bias visually with the funnel plot or with Egger's test.

Meta‐analysis 3: Do remote intervention(s) evaluated as a replacement or partial replacement for in‐person treatment reduce relapse compared to exclusively in‐person treatment?

People who received a remote intervention as a replacement or partial replacement for in‐person treatment were 49% less likely to relapse compared to those who received exclusively in‐person treatment (OR = 0.51; 95% CI = 0.34, 0.76; *P* = 0.001; 7 interventions; 1062 participants; I^2^ = 39.7%) [see Figure 4(c)]. The relatively wide CI is likely to be affected by findings from Gonzales et al. [61], which appears an outlier. RoB was judged to be ‘high’ or of ‘some concerns’ across most outcomes, with ‘low’ in two outcomes. Tests for publication bias were underpowered for this meta‐analysis, although visual inspections of the funnel plot indicated asymmetry.

Meta‐analysis 4: Do remote interventions evaluated as a replacement or partial replacement for in‐person treatment reduce days of alcohol/drug use compared to exclusively in‐person treatment?

There was a reduction in days of alcohol/drug use in people who received remote interventions as a replacement or partial replacement for in‐person treatment when compared with exclusively in‐person treatment, but this finding was uncertain (SMD = −0.08; 95% CI = −0.24 to 0.07; *P* = 0.301; 8 interventions; 1610 participants; I^2^ = 48.4%) [see Figure 4(d)]. RoB was judged to be ‘high’ or of ‘some concerns’ across most outcomes, with ‘low’ in one outcome. Tests for publication bias were underpowered for this meta‐analysis, although there were no indications of publication bias visually or in the results of Egger's test.

### Subgroup analyses

For remote interventions supplementing in‐person treatment, we conducted four subgroup analyses. We were unable to perform subgroup analysis for remote interventions replacing/partially replacing in‐person treatment because there were too few studies. The subgroup analyses examined whether effects for each outcome varied according to the substance targeted and the therapeutic approach (remote recovery support, remote talking therapy and self‐guided therapy). They showed mixed effects, with some subgroups containing small numbers or single studies, and were inconclusive, as summarised in Table 5.

**TABLE 5:** Summary of subgroup analyses by intervention type and type of substance.

Outcomes		
Intervention type	Relapse	Days of use
SGT	OR = 0.31 (95% CI = 0.10, 0.91), 5 interventions, I^2^ = 69.6% (substantial)	SMD = −0.23 (95% CI = −0.39 to −0.07), 12 interventions, I^2^ = 35.4%
RRS	OR = 0.76 (95% CI = 0.58, 0.99), 5 interventions, I^2^ = 0%	SMD = −0.10 (95% CI = −0.22 to 0.02), 4 interventions, I^2^ = 0%
RTT	OR = 0.69 (95% CI = 0.48, 0.97), 6 interventions, I^2^ = 0%	SMD = −0.14 (95% CI = −0.44 to 0.17), 1 intervention, I^2^ = 0%
Game	OR = 1.25 (95% CI = 0.34, 4.56), 1 intervention, I^2^ = 0%	
RRS + RTT	OR = 0.64 (95% CI = 0.20, 2.09), 1 intervention, I^2^ = 0%	
**Type of substance**		
Alcohol	OR = 0.70 (95% CI = 0.55, 0.80), 11 interventions, I^2^ = 0%	SMD = 0.00 (95% CI = −0.17 to 0.17), 4 interventions, I^2^ = 0%
Drugs and alcohol	OR = 0.67 (95% CI = 0.43, 1.04), 4 interventions, I^2^ = 0%	SMD = −0.22 (95% CI = −0.36 to −0.08), 10 interventions, I^2^ = 35.7%
Drugs	OR = 0.44 (95% CI = 0.08, 2.48), 2 interventions, I^2^ = 93.6% (substantial)	SMD = −0.25 (95% CI = −0.46 to −0.04), 3 interventions, I^2^ = 4.2%

Abbreviations: I^2^, over 50% = substantial heterogeneity; RRS, remote recovery support; RTT, remote talking therapy; SGT, self‐guided therapy.

## DISCUSSION

### Summary of evidence

We meta‐analysed 42 interventions from 34 RCTs. Pooled effect estimates showed a 39% lower odds of relapse (95% CI = 0.46, 0.81) and a reduction in the mean days of alcohol/drug use (SMD = −0.18; 95% CI = −0.28 to −0.08) at the end of intervention for people receiving a remote intervention plus in‐person treatment, when compared to those who received in‐person treatment alone. This finding suggests that supplementing in‐person treatment with a remote intervention could be an effective approach for achieving positive treatment outcomes. The pooled effect estimates for replacing or partially replacing in‐person treatment with remote intervention(s) were less conclusive. Analyses showed the odds of relapse among the intervention group was approximately half that of the control group (OR = 0.51), but only a very small and uncertain reduction in days of use (SMD = −0.08; 95% CI = −0.24 to 0.07; *P* = 0.301). The conclusiveness of these latter findings is undermined by the smaller pool of evidence and the uncertainty of the days of use finding. Furthermore, although heterogeneity (I^2^) was moderate across the four main meta‐analyses and did not rise above 50% (our pre‐specified threshold for substantial heterogeneity), the finding for replacement or partial replacement interventions on days of use was close to that threshold (I^2^ = 48.4%). Additionally, because the assumption was that the replacement and partial replacement interventions should not result in worse outcomes than in‐person treatment, the large effect on relapse was unexpected. We adopted several approaches to try to identify an explanation for this surprising finding. First, one study was an outlier in terms of its large effect size [61], but a sensitivity analysis in which this study was removed did not result in a major change in the pooled estimate (OR = 0.61; 95% CI = 0.47, 0.81). Second, we scrutinised each intervention in this analysis. This revealed that the intervention with the greatest weight in the analysis [60] was perhaps more like a supplement to in‐person treatment than a replacement. The intervention was designed to partially replace in‐person treatment: ‘a substitute for approximately 2 hours of clinician time’. However, the authors note in their discussion that although patients in the intervention condition ‘were assigned to attend fewer standard care sessions’ in practice they ‘ended up attending a similar number of usual counselling sessions as the control patients’ (page 8), meaning that they experienced a higher overall dose of therapy than controls. Further, although this study was the only included study assessed to be at low risk of bias, the external generalizability of the findings has been called into question as the sample was found to be not well‐representative of the target population [67]. We conducted a further sensitivity analysis, exploring the impact of removing Campbell et al. [60], but this reinforced the large effect on relapse (OR = 0.45; 95% CI = 0.26, 0.79). We were unable to identify any other explanation for the large pooled effect.

Subgroup analyses to explore whether effects of remote interventions varied according to substance and therapeutic approach showed mixed effects and were inconclusive. Approximately 70% of the outcomes were judged to be of high RoB, therefore, all findings should be interpreted with caution.

Overall, our findings are in line with the meta‐analysis previously discussed, that showed in six RCTs a decrease in opioid and stimulant use among users receiving digital community reinforcement and contingency management as a supplement to in‐person support when compared to controls [21]. Another systematic narrative review of 20 RCTs reported mixed findings with half the studies showing differences in abstinence between digital intervention groups and controls for opioid users [68]. The remote interventions included in our review were heterogeneous, diverse and complex. Their content, frequency, duration, types, settings, professional/paraprofessional support and fidelity of delivery varied considerably. There were also wide variations in the range of control conditions. The dynamic interplay of these elements is likely to have substantial influence acting as moderators and/or mediators on the mechanism and impact of the interventions for different populations. This made synthesis and comparison between studies challenging, and these challenges had also been experienced by other reviewers [68, 69, 70].

Study participants were predominately male. Although not the focus of this review, it is apparent that certain populations were under‐represented in the included studies (e.g. women, ethnic minorities and people with mental health problems). As part of the wider review we explored whether there is a differential impact of remote interventions in relation to different population characteristics, their life situation and their context [22]. We also conducted a QCA to identify what combinations of features of remote interventions were associated with the most and least effectiveness [22].

### Strengths and limitations

The current work is the first comprehensive attempt to synthesize existing evidence on the effectiveness of remote interventions to support alcohol/drug treatment, as far as we are aware. We followed robust methods according to PRISMA.

Previous reviews combined different types of remote or digital interventions, regardless of whether they were aimed at prevention, early intervention or treatment, whether they evaluated remote therapies as a supplement to or replacement for in‐person treatment, or whether they involved self‐guided therapy, remote talking therapy or remote recovery support [70, 71, 72, 73]. We focused exclusively on treatment, differentiated between evaluation types and explored the effects of specific therapeutic approaches.

We included a sizable evidence base of 34 RCTs reporting 49 outcomes in 42 interventions. We included all six of the studies meta‐analysed in the only previously published systematic review focused on a treatment population [21], as well as 28 other studies.

Nevertheless, there were limitations in this review. Although our search strategy was broad and extensive, it is possible that not all relevant studies were captured. It excluded potential eligible studies undertaken in non‐OECD countries and published in languages other than English. The evidence was dominated by studies conducted in the United States, potentially limiting the applicability of the findings to other settings.

We used the RoB 2 tool to assess RoB at the outcome level within each RCT, rather than the RCT as a whole. This robust method enabled us to present a more accurate and precise assessment of bias highlighting the potential impact it had on the strength of the evidence. Only one study was classified as having a low RoB in all the domains. The most prevalent type of RoB related to the use of self‐report outcome measures (domain 3), particularly for the ‘days of use’ outcome, and missing outcome data (domain 4). These concerns have been noted by others, who recognise that such issues are inherent in evaluations of alcohol/drug interventions [74, 75, 76]. Outcomes comprising retrospective self‐reported use can be susceptible to under‐reporting because of recall bias and social desirability bias. It is also the case, that when the interventions themselves involved regular monitoring/reminders, participants were likely to have a more accurate recall of alcohol/drug use than the control group. We were, therefore, more confident about the estimates of effect for the toxicology‐verified outcomes of relapse.

It has previously been acknowledged that attrition rates are typically high in addiction treatment research studies [77], likewise with remote interventions [74, 78]. In the included studies, attrition rates were possibly high because of low level of adherence to on‐line interventions involving minimal human interaction. Conversely, the anonymity offered by on‐line interventions could also encourage engagement and program completion. Nevertheless, the missingness of data because of attrition, the rate of attrition and the way attrition was dealt with in the analyses are likely to influence the true values of the findings.

Other RoB 2 domains can also present challenges for alcohol/drug treatment interventions. ‘Blinding’ of participants can be difficult as the nature of the interventions often required the participants to be trained or instructed on how to use and access them, therefore, it was likely that participants would have been aware of the interventions they were receiving. Pre‐registered protocols with analysis plans were commonly lacking, as noted by others [76], and we were often unable to establish data analysis intentions. The RoB 2 assessments highlighted these weaknesses, which must be considered when interpreting the strength of the evidence and its impact on the integrity, applicability and generalizability of our findings.

We also noted several other key limitations. Usable data for our meta‐analysis were not available from all of the potentially eligible RCTs identified, because of inconsistency and/or incomplete outcome data reported (e.g. lack of Standards Deviations). The lack of standardisation of measures used to assess core outcomes such as abstinence and alcohol/drug use rendered comparison between a larger number of eligible studies difficult. As follow‐up data reported post‐intervention was limited, we assessed outcomes at the nearest endpoint to the end of the interventions. This prevented us from exploring whether the benefits of remote interventions could be sustained in the longer term.

### Implications for practice and research

Remote interventions can offer many advantages such as improving access, convenience, opportunities for self‐management, interactive contact between patients and care providers, and reducing social and geographical barriers. Findings from this review provide some evidence for the general efficacy of remote and digital interventions in supporting alcohol/drug treatment, either as a supplement to in‐person treatment or as a replacement or partial replacement for in‐person treatment. However, most of the evidence is at high risk of bias and so findings should be interpreted with caution.

The development of a list of standard outcome criteria by the research community would be most useful to aid future alcohol/drug research in enabling synthesis of a larger body of evidence. Design of future studies should consider and address some of the potential sources of bias and limitations of studies experienced in our review. Treatment and recovery is a lengthy process, and studies with follow‐up periods beyond the intervention's end, to evaluate long‐term effects, would be useful.

Given the rapid pace at which technology is evolving, more new studies evaluating digital interventions for alcohol/drug misuse are being published. Our update search in 2023 identified five additional RCTs. Applications (apps) that run on mobile or desktop devices [79] have become integral to everyday life and are regularly used. However, only three of the studies included in this review were delivered through apps. Evidence for app‐based interventions in alcohol/drug treatment would be of great research interest.

## CONCLUSIONS

The digital health landscape is expanding and evolving at speed, presenting unique opportunities to increase and expand reach, uptake and engagement of remote/digital interventions to support alcohol/drug treatment. Remote interventions delivered as a supplementary component of in‐person alcohol/drug treatment appear to be an effective approach to reducing the likelihood of relapse and days of alcohol/drug use. The evidence is not conclusive on replacing, or partially replacing in‐person treatment with remote interventions, but it does not appear to lead to worse outcomes. However, because much of the evidence is at high risk of bias, these findings should be interpreted with caution. The next challenges lie in exploring which features and combinations of intervention components are associated with the most effective remote interventions and understanding for whom such configurations can work to bring real benefit.

## AUTHOR CONTRIBUTIONS

**Irene Kwan:** Conceptualization (supporting); data curation (equal); formal analysis (supporting); investigation (equal); methodology (equal); project administration (supporting); software (supporting); supervision (supporting); validation (equal); visualization (equal); writing—original draft (lead); writing—review and editing (equal). **Helen E. D. Burchett:** Conceptualization (lead); data curation (equal); formal analysis (lead); funding acquisition (lead); investigation (equal); methodology (equal); project administration (lead); software (supporting); supervision (lead); validation (equal); visualization (equal); writing—original draft (lead); writing—review and editing (equal). **Wendy Macdowall:** Conceptualization (supporting); data curation (equal); formal analysis (lead); funding acquisition (supporting); investigation (equal); methodology (equal); project administration (supporting); software (supporting); supervision (supporting); validation (equal); visualization (equal); writing—original draft (supporting); writing—review and editing (equal). **Preethy D'Souza:** Conceptualization (supporting); data curation (equal); formal analysis (lead); funding acquisition (supporting); investigation (equal); methodology (equal); project administration (supporting); software (supporting); supervision (supporting); validation (equal); visualization (equal); writing—original draft (supporting); writing—review and editing (equal). **Claire Stansfield:** Conceptualization (supporting); data curation (equal); funding acquisition (supporting); investigation (equal); methodology (equal); project administration (supporting); software (lead); supervision (supporting); writing—original draft (supporting); writing—review and editing (equal). **Dylan Kneale:** Conceptualization (supporting); data curation (equal); formal analysis (lead); funding acquisition (lead); investigation (equal); methodology (equal); project administration (supporting); software (supporting); supervision (supporting); validation (equal); visualization (equal); writing—original draft (supporting); writing—review and editing (equal). **Katy Sutcliffe:** Conceptualization (lead); data curation (equal); formal analysis (lead); funding acquisition (lead); investigation (equal); methodology (equal); project administration (lead); software (supporting); supervision (lead); validation (equal); visualization (equal); writing—original draft (lead); writing—review and editing (equal).

## DECLARATION OF INTERESTS

None.

## STUDY REGISTRATION

PROSPERO CRD42022339953.

## Supporting information


**Appendix 1:** PRISMA abstract checklist.

**Appendix 2:** PRISMA review checklist.

**Appendix 3:** Medline search strategy.

**Appendix 4:** Approaches to screening using priority screening and machine classifiers.

**Appendix 5:** Data extraction (coding) tool.

**Appendix 6:** Risk‐of‐Bias 2 assessments according to outcomes (n=34 RCTs, 42 interventions).

## Data Availability

The data that support the findings of this study are available from the corresponding author upon reasonable request.
