# Supplementary material for: How effective are remote and/or digital interventions as part of alcohol and drug treatment and recovery support? A systematic review and meta‐analysis
Source: Addiction. 2025 Mar 24;120(8):1531–50. doi: 10.1111/add.70021 (PMC12215248; doi:10.1111/add.70021)
Supplement: Supplementary file 3 — Appendix 3: Medline search strategy. [file ADD-120-1531-s003.docx]

**APPENDIX 3 Medline search strategy**

**MEDLINE Search 14 July 2023**

This search consists of i) update searches from the earlier map (line 57) and ii) an update search from the expanded scope of the review (line 87).

Saved records from lines 57 and 87= 12,676

**Database: Ovid MEDLINE(R) ALL <1946 to July 13, 2023>**
**Search Strategy:**

**1**  (Substance adj2 ("use" or user* or usage or misuse or abuse* or misuse or depend* or addict* or disorder*)).ti,ab,kw. (77906)
**2**  ((solvent* or drug or drugs) adj3 (addict* or abus* or misuse* or user or users or disorder* or dependen* or recovery or intoxicat* or withdraw* or detox* or habit* or recreation* or illicit or relapse)).ti,ab,kw. (114259)
**3**  ((cocaine or marijuana* or cannab* or hashish or opium or opioid* or opiate* or heroin or amphetamine* or methamphetamine* or Ketamine or ecstasy or MDMA or "recreational drugs" or "illicit drugs" or "illicit substances" or "street drug" or "street drugs" or "poly-drug" or polydrug or morphine or meth or methadone or methoxetamine) adj2 (addict* or abus* or misuse* or user or users or disorder* or dependen* or recovery or intoxicat* or withdraw* or detox* or habit* or "use" or abstain* or abstinence or relapse or craving)).ti,ab,kw. (93901)
**4**  ("club drug" or "club drugs" or "Drug using population" or "Drug using populations" or "who inject drugs" or "who use drugs").ti,ab,kw. (6204)
**5**  "Drug problems".ti,ab,kw. (1128)
**6**  "drug use".ti,ab,kw. (53172)
**7**  "drug treatment".ti,ab,kw. (34951)
**8**  "drug prevention".ti,ab,kw. (777)
**9**  Street drugs/ or crack cocaine/ or designer drugs/ or substance-related disorders/ or Amphetamine-Related Disorders/ or cocaine-related disorders/ or inhalant abuse/ or marijuana abuse/ or opioid-related disorders/ or heroin dependence/ or morphine dependence/ or opium dependence/ or substance abuse, intravenous/ or phencyclidine abuse/ or substance abuse, oral/ or exp substance withdrawal syndrome/ or drug users/ (189641)
**10**  Illicit Drugs/ or exp Narcotic-Related Disorders/ or Alcoholics Anonymous/ (47336)
**11**  exp Alcohol-related disorders/ or alcoholism/ or alcoholics/ or binge drinking/ or alcohol abstinence/ or alcohol intoxication/ or exp Alcohol-Induced Disorders/ or Wernicke Encephalopathy/ or Alcohol Withdrawal Delirium/ or Alcohol Withdrawal Seizures/ or Psychoses, Substance-Induced/ (127034)
**12**  alcohol drinking/th (775)
**13**  (Alcoholic* or alcoholism).ti,ab,kw. (99910)
**14**  (alcohol* adj3 (abus* or misuse* or disorder* or problem* or dependen* or treatment* or recovery or quit* or anonymous or harmful* or hazardous or intoxicat* or risky* or withdraw* or detox* or heavy or heavily or excess* or therap* or habit* or addict* or unhealthy or abstinence)).ti,ab,kw. (99842)
**15**  (((Drinker* or drinking) adj2 (binge or risky or excess* or harmful* or hazardous* or heavy or heavily or unhealthy)) or (Alcohol and (drink* adj2 problem*))).ti,ab,kw. (23112)
**16**  ((("at risk" or relapse* or risky) adj2 drink*) or (risk adj2 drinker*)).ti,ab,kw. (2675)
**17**  (((risky or unhealthy or harmful or excess* or heavy or hazardous) adj1 consumption) and alcohol).ti,ab,kw. (986)
**18**  1 or 2 or 3 or 4 or 5 or 6 or 7 or 8 or 9 or 10 or 11 or 12 or 13 or 14 or 15 or 16 or 17 (557895)
**19**  Computer Terminals/ or Microcomputers/ or minicomputers/ or Computers, Handheld/ or Smartphone/ or Telemedicine/ or Telerehabilitation/ or Mobile applications/ or Text messaging/ or Cell phone/ or Therapy, computer assisted/ or Information technology/ or Internet/ or speech recognition software/ or Computer simulation/ or virtual reality/ or User-computer interface/ or Social networking/ or online social networking/ or "cell phone use"/ or Technology transfer/ or internet access/ or Virtual reality exposure therapy/ or automation/ or social media/ or computer communication networks/ or Wireless technology/ or telecommunications/ or Telemetry/ or Remote Sensing Technology/ or Wearable electronic devices/ or medical informatics applications/ (476360)
**20**  Smart Glasses/ or "Internet-Based Intervention"/ or Ambient Intelligence/ or "Internet use"/ (1891)
**21**  (therapy or "prevention and control" or rehabilitation).fs. (2316951)
**22**  User-computer interface/ or treatment outcome/ or Telemedicine/ or Telerehabilitation/ or Precision medicine/ or patient care/ or rehabilitation/ or self care/ or Therapy, computer assisted/ or Secondary prevention/ or Primary prevention/ or Tertiary prevention/ or Self help groups/ or Feedback, Psychological/ or Feedback, Sensory/ or Biofeedback, Psychology/ or Behavior Therapy/ or Neurofeedback/ or Mind-Body Therapies/ or Psychotherapy/ or Psychosocial Support Systems/ or Social Support/ or "Treatment Adherence and Compliance"/ or "Ecological Momentary Assessment"/ or Behavior control/ or risk reduction behavior/ or evaluation studies as topic/ or pilot projects/ or feasiblity studies/ or program evaluation/ or benchmarking/ or Health Behavior/ or health risk behaviors/ or Feedback/ or Harm reduction/ or Patient Education as Topic/ (1977494)
**23**  "Duration of Therapy"/ or "Self-Testing"/ or Alcoholics Anonymous/ or exp "Mind-Body Therapies"/ or exp "social support"/ (128929)
**24**  21 or 22 or 23 (3837048)
**25**  (19 or 20) and 24 (127521)
**26**  ((intervention* or program* or service*) and (mobile or web* or computer* or digital* or wireless* or Bluetooth or cyber* or online* or virtual* or intelligen* or software or Cellular phone* or cell phone* or electronic* or smartphone* or "smart phone" or "smart phones" or automated or electronic* or (portable adj2 media) or Internet* or Technolog* or Automation or microcomp* or ipad or iphone or ipod or netbook or "touch screen" or hardware or software or "multimedia device" or "multi media device" or "portable device*" or ("hand held" adj2 device*) or (handheld adj2 device*) or texting* or "text messag*" or SMS or ("short messag*" adj1 service*) or (text adj3 deliver*) or "social networking" or "social media" or Messenger* or Facebook or Whatsapp)).ti. (29187)
**27**  ((intervention* or program* or service*) adj5 (mobile or web* or computer* or digital* or wireless* or Bluetooth or cyber* or online* or virtual* or intelligent* or Cellular phone* or cell phone* or electronic* or smartphone* or "smart phone" or "smart phones" or automated or (artificial adj2 intelligen*) or (portable adj2 media) or Internet* or Technolog* or Automation or microcomp* or ipad or iphone or ipod or netbook or "touch screen" or hardware or software or "multimedia device" or "multi media device" or "portable device*" or ("hand held" adj2 device*) or (handheld adj2 device*) or texting* or "text messag*" or SMS or ("short messag*" adj1 service*) or (text adj3 deliver*) or "social networking" or "social media" or Messenger* or Facebook or Whatsapp)).ab. (94383)
**28**  ("mhealth" or "mobile health" or "m health" or "e health" or ehealth or ("electronic health" not "electronic health record*")).ti,ab. (17262)
**29**  (telehealth* or telemedicine or teletherap* or "tele health*" or "tele medicine" or "tele therap*" or telemonitor* or "tele monitor*").ti,ab. (31401)
**30**  (smartwatch* or "smart watch" or "smart watches" or "smart shoe*" or "smart book*" or "assistive technolog*" or (digital* adj2 phenoty*) or "Augmented Reality" or "Virtual Reality").ti,ab. (23527)
**31**  ((smart* or wearable) adj3 (device or technolog* or sensor* or track*)).ti,ab. (16491)
**32**  (voice adj2 (response or recog* or automat* or intelligent* or electronic* or Internet or computer* or digital*)).ti,ab. (2260)
**33**  (mobile-sensing or "mobile sensing" or msens* or geosens* or geolocat* or geofenc* or "geo sens*" or "geo-sens*" or "geo fenc*" or "geo-fenc*" or "geo locat*" or "geo-locat*" or Ecounsel* or eCBT or "e CBT" or etherapy or "e therapy" or "eSBI" or "e SBI" or chatroom* or "chat room*" or (text adj3 chat*) or chatbot* or "live chat*" or "chat bot" or "chat bots" or "chat interface*" or "chat forum*" or "chat site" or "chat sites" or chatsite* or chatbox* or "chat box*" or breathal*).ti,ab. (3825)
**34**  (app or apps or "app-based").ti. (11528)
**35**  (((smartphone* or "smart phone" or "smart phones") adj3 apps) or (mobile* adj3 apps) or (digital* adj3 apps) or (electronic* adj3 apps) or (web* adj3 apps) or (internet* adj3 apps) or (computer* adj3 apps)).ab. (4885)
**36**  (((smartphone* or "smart phone" or "smart phones") adj3 app) or (mobile* adj3 app) or (digital* adj3 app) or (electronic* adj3 app) or (web* adj3 app) or (internet* adj3 app) or (computer* adj3 app)).ab. (6994)
**37**  (((smartphone* or "smart phone" or "smart phones") adj3 application*) or (mobile* adj3 application*) or (digital* adj3 application*) or (electronic* adj3 application*) or (web* adj3 application*) or (internet* adj3 application*) or (computer* adj3 application*)).ti,ab. (31757)
**38**  (((Device* or platform* or interface* or deliver* or assist* or facilitat* or guid* or aid* or generat* or application*) adj3 (portable or mobile* or web* or computer* or digital* or wireless or Bluetooth or cyber* or online or virtual* or intelligen* or software or Cellular phone* or cell phone* or electronic* or automated or smartphone* or "smart phone" or "smart phones" or electronic* or "multi media" or multimedia or handheld or "hand held" or Internet* or Technolog* or Automation or microcomp* or ipad* or iphone* or ipod* or netbook* or "touch screen" or hardware or software)) and ((behav* adj2 chang*) or support* or treatment* or feedback or monitor* or chat* or interact* or advice or advis* or tailor* or personalis* or counsel* or therap* or "self help" or "self-help" or "self care" or "self-care" or "self-guide*" or "self guide*" or communicat* or messag* or biofeedback or rehab* or "momentary assessment*" or "momentary intervention*" or "mutual help" or prevent* or forum or discuss* or comment* or post* or share or sharing or network*)).ti,ab. (179031)
**39**  (((behav* adj2 chang*) or support* or treatment* or feedback or monitor* or chat* or interact* or advice or advis* or tailor* or personalis* or counsel* or therap* or "self help" or "self-help" or "self care" or "self-care" or "self-guide*" or "self guide*" or communicat* or messag* or biofeedback or rehab* or "momentary assessment" or "momentary intervention*" or "mutual help" or prevent*) adj5 (portable or mobile* or web* or computer* or digital* or wireless or Bluetooth or cyber* or online or virtual* or intelligen* or software or cellular phone* or cell phone* or electronic* or automated or smartphone* or "smart phone" or "smart phones" or electronic* or "multi media" or multimedia or handheld or "hand held" or Internet* or technolog* or automation or microcomp* or ipad* or iphone* or ipod* or netbook* or "touch screen" or hardware or software)).ti,ab. (201473)
**40**  ((forum or discuss* or comment* or post* or share or sharing or network*) adj5 (portable or mobile* or web* or computer* or digital* or wireless or Bluetooth or cyber* or online or virtual* or intelligen* or software or cellular phone* or cell phone* or electronic* or automated or smartphone* or "smart phone" or "smart phones" or electronic* or "multi media" or multimedia or handheld or "hand held" or Internet* or technolog* or automation or microcomp* or ipad* or iphone* or ipod* or netbook* or "touch screen" or hardware or software)).ti,ab. (74125)
**41**  (assessment adj5 (web* or computer* or online or virtual* or electronic* or automated or Internet* or text*)).ti,ab. (14152)
**42**  ((texting* or "text messag*" or SMS or ("short messag*" adj1 service*) or (text adj3 deliver*) or "social networking" or "social media" or Messenger* or Facebook or Whatsapp) adj5 ((behav* adj2 chang*) or support* or treatment* or feedback or monitor* or chat* or interact* or advice or advis* or tailor* or personalis* or counsel* or therap* or "self help" or "self-help" or "self care" or "self-care" or "self-guide*" or "self guide*" or biofeedback or rehab* or "momentary assessment" or "momentary intervention*" or "mutual help" or prevent*)).ti,ab. (7697)
**43**  ((portable or mobile or web* or computer* or digital* or wireless or Bluetooth or cyber* or online or virtual* or intelligen* or software or Cellular phone* or cell phone* or electronic* or automated or smartphone* or "smart phone" or "smart phones" or electronic* or "multi media" or multimedia or handheld or "hand held" or Internet* or Technolog* or Automation or microcomp* or ipad or iphone or ipod or netbook or "touch screen" or hardware or software or texting* or "text messag*" or SMS or ("short messag*" adj1 service*) or (text adj3 deliver*) or "social networking" or "social media" or Messenger* or Facebook or Whatsapp) and (recovery or relapse or withdraw* or abstinence)).ti. (1785)
**44**  ((portable or mobile or web* or computer* or digital* or wireless or Bluetooth or cyber* or online or virtual* or intelligen* or software or Cellular phone* or cell phone* or electronic* or automated or smartphone* or "smart phone" or "smart phones" or electronic* or "multi media" or multimedia or handheld or "hand held" or Internet* or Technolog* or Automation or microcomp* or ipad or iphone or ipod or netbook or "touch screen" or hardware or software or texting* or "text messag*" or SMS or ("short messag*" adj1 service*) or (text adj3 deliver*) or "social networking" or "social media" or Messenger* or Facebook or Whatsapp) and ((reduc* or increase* or frequency or prevent* or curb*) adj3 (intake or consumption or alcohol or drink* or drug or drugs or "substance use" or substances or illicit or solvent or cocaine or marijuana* or cannab* or hashish or opium or opioid* or opiate* or heroin or amphetamine* or methamphetamine* or Ketamine or ecstasy or MDMA or "poly-drug" or polydrug or morphine or meth or methadone or methoxetamine))).ti. (548)
**45**  26 or 27 or 28 or 29 or 30 or 31 or 32 or 33 or 34 or 35 or 36 or 37 or 38 or 39 or 40 or 41 or 42 or 43 or 44 (548113)
**46**  25 or 45 (610070)
**47**  18 and 46 (11619)
**48**  limit 47 to yr="2004 -Current" (10447)
**49**  animals/ not (animals/ and humans/) (5103579)
**50**  48 not 49 (10296)
**51**  limit 50 to (comment or editorial) (95)
**52**  50 not 51 (10201)
**53**  limit 52 to english language (9973)
**54**  limit 52 to (afrikaans or albanian or arabic or armenian or azerbaijani or belorussian or bengali or bosnian or bulgarian or burmese or catalan or chinese or croatian or czech or danish or dutch or esperanto or estonian or finnish or flemish or french or gaelic, scots or georgian or german or greek or hausa or hebrew or hindi or hungarian or icelandic or indonesian or interlingua or italian or japanese or kirghiz or korean or latin or latvian or lithuanian or macedonian or malay or marathi or masai or multilingual or norwegian or persian or polish or portuguese or pushto or rumanian or russian or serbian or slovak or slovene or spanish or swahili or swedish or tagalog or tamil or telugu or thai or turkish or ukrainian or undetermined or urdu or vietnamese or welsh) (271)
**55**  53 or 54 (10201)
**56**  53 and 54 (43)
**57**  53 or 56 (9973)

**61**  (phone or phones or cellphone* or telephon* or video or videoconf* or videotelephon* or videophone* or telepresence or voip or skype or zoom or facetime* or "hangouts" or "google duo" or "talky core" or viber or tango or zoom or wechat or oovoo or Justalk or "Microsoft teams" or groupware or telecom* or "tele comm*" or telecounsel* or "tele counsel*" or telepsych* or "tele psyc*" or "video counsel*" or "video doctor*" or "video therap*" or avatar*).ti,ab,kw. (244169)
**62**  (remote* adj2 (commun* or deliver* or platform* or system* or administer* or support or feedback or messag* or advice or interact* or adviso* or service* or guid* or "self-guid*" or assist* or monitor* or chat* or tailor* or personal* or facilitate* or aid or aided)).ti,ab,kw. (15122)
**63**  (Treatment or treating or counsel* or consultation* or management or therap* or intervention* or "self-help" or "self manag*" or "self care" or "self-guide*" or keywork or "key work" or keyworking or "keyworking" or groupwork or "group work" or "mutual aid" or "mutual help" or "psychology session*" or adherence or psychotherap* or program* or service* or Recovery or rehab* or detox* or withdraw* or (relapse adj3 prevent*) or (abstinence adj3 maint*) or (Transition* adj2 (support* or help*)) or "Motivational Interview*" or monitoring or Reinforcement or Aftercare or "after care" or "follow up care" or (follow* adj2 care) or (Support adj2 (group or forum or network or forums or groups or networks or peer or peers)) or Postdischarg* or discharge* or outpatient* or patient* or "Primary care" or "secondary care" or clinic or "momentary assessment" or biofeedback or (delivery adj3 care) or (delivery adj3 healthcare) or (telecounsel* or "tele counsel*" or telepsych* or "tele psyc*" or "video counsel*" or "video doctor*" or "video therap*")).ti,ab,kw. (14432168)
**64**  telephone/ or answering services/ or videoconferencing/ or distance counseling/ (15629)
**65**  remote consultation/ or distance counseling/ (5810)
**66**  "delivery of health care"/ or after-hours care/ or "delivery of health care, integrated"/ or health services accessibility/ (211210)
**67**  aftercare/ or rehabilitation/ or transitional care/ (32764)
**68**  User-computer interface/ or treatment outcome/ or Telemedicine/ or Telerehabilitation/ or Precision medicine/ or patient care/ or rehabilitation/ or self care/ or Therapy, computer assisted/ or Secondary prevention/ or Tertiary prevention/ or exp Self help groups/ or Feedback, Psychological/ or Feedback, Sensory/ or Biofeedback, Psychology/ or Behavior Therapy/ or Neurofeedback/ or exp Mind-Body Therapies/ or Psychotherapy/ or Psychosocial Support Systems/ or exp Social Support/ or "Treatment Adherence and Compliance"/ or "Ecological Momentary Assessment"/ or evaluation studies as topic/ or pilot projects/ or feasiblity studies/ or program evaluation/ or benchmarking/ or Health Behavior/ or health risk behaviors/ or Feedback/ or Harm reduction/ or Patient Education as Topic/ or Motivational Interviewing/ or "Duration of Therapy"/ or "Self-Testing"/ (1983429)
**69**  (therapy or rehabilitation).fs. (2316951)
**70**  (remote* and (Treatment or treating or counsel* or consultation* or management or therap* or intervention* or "self-help" or "self manag*" or "self care" or "self-guide*" or keywork or "key work" or keyworking or "keyworking" or groupwork or "group work" or "mutual aid" or "mutual help" or "psychology session*" or adherence or psychotherap* or program* or service* or Recovery or rehab* or detox* or withdraw* or (relapse adj3 prevent*) or (abstinence adj3 maint*) or (Transition* adj2 (support* or help*)) or "Motivational Interview*" or monitoring or Reinforcement or Aftercare or "after care" or "follow up care" or (follow* adj2 care) or (Support adj2 (group or forum or network or forums or groups or networks or peer or peers)) or Postdischarg* or discharge* or outpatient* or patient* or "Primary care" or "secondary care" or clinic or "momentary assessment" or biofeedback or (delivery adj3 care) or (delivery adj3 healthcare))).ti. (8009)
**71**  (remote* adj5 (Treatment or treating or counsel* or consultation* or management or therap* or intervention* or "self-help" or "self manag*" or "self care" or "self-guide*" or keywork or "key work" or keyworking or "keyworking" or groupwork or "group work" or "mutual aid" or "mutual help" or "psychology session*" or adherence or psychotherap* or program* or service* or Recovery or rehab* or detox* or withdraw* or (relapse adj3 prevent*) or (abstinence adj3 maint*) or (Transition* adj2 (support* or help*)) or "Motivational Interview*" or monitoring or Reinforcement or Aftercare or "after care" or "follow up care" or (follow* adj2 care) or (Support adj2 (group or forum or network or forums or groups or networks or peer or peers)) or Postdischarg* or discharge* or outpatient* or patient* or "Primary care" or "secondary care" or clinic or "momentary assessment" or biofeedback or (delivery adj3 care) or (delivery adj3 healthcare))).ti. (5914)
**72**  ((Substance adj2 ("use" or user* or usage or misuse or abuse* or misuse or depend* or addict* or disorder*)) or ((solvent* or drug or drugs) adj3 (addict* or abus* or misuse* or user or users or disorder* or dependen* or recovery or intoxicat* or withdraw* or detox* or habit* or recreation* or illicit or relapse)) or ((cocaine or marijuana* or cannab* or hashish or opium or opioid* or opiate* or heroin or amphetamine* or methamphetamine* or Ketamine or ecstasy or MDMA or "recreational drugs" or "illicit drugs" or "illicit substances" or "street drug" or "street drugs" or "poly-drug" or polydrug or morphine or meth or methadone or methoxetamine) adj2 (addict* or abus* or misuse* or user or users or disorder* or dependen* or recovery or intoxicat* or withdraw* or detox* or habit* or "use" or abstain* or abstinence or relapse or craving)) or ("club drug" or "club drugs" or "Drug using population" or "Drug using populations" or "who inject drugs" or "who use drugs") or "Drug problems" or "drug use").ti,ab,kw. or Street drugs/ or crack cocaine/ or designer drugs/ or substance-related disorders/ or Amphetamine-Related Disorders/ or cocaine-related disorders/ or inhalant abuse/ or marijuana abuse/ or opioid-related disorders/ or heroin dependence/ or morphine dependence/ or opium dependence/ or substance abuse, intravenous/ or phencyclidine abuse/ or substance abuse, oral/ or exp substance withdrawal syndrome/ or drug users/ or Illicit Drugs/ or exp Narcotic-Related Disorders/ or Alcoholics Anonymous/ (342637)
**73**  ((alcohol* adj3 (abus* or misuse* or disorder* or dependen* or treatment* or treating or recovery or therap* or addict* or problem* or hazardous or withdraw* or detox*)) or (Recovery adj3 (drinking or alcohol* or drinkers))).ti,ab,kw. or Alcohol/ or exp Alcohol-related disorders/ or alcoholism/ or alcoholics/ or exp Alcohol-Induced Disorders/ or alcohol drinking/th or ((Alcoholic* not ((alcoholic adj1 beverage*) or (alcoholic adj1 drink) or (alcoholic adj1 drinks))) or alcoholism).ti,ab,kw. (200622)
**74**  72 or 73 (503270)
**75**  61 or 62 or 64 or 65 (263501)
**76**  63 or 65 or 66 or 67 or 68 or 69 (15404366)
**77**  75 and 76 (184965)
**78**  70 or 71 (8009)
**79**  77 or 78 (189243)
**80**  74 and 79 (4682)
**81**  limit 80 to yr="2004 -Current" (3950)
**82**  81 not 49 (3923)
**83**  limit 82 to english language (3842)
**84**  limit 82 to (afrikaans or albanian or arabic or armenian or azerbaijani or belorussian or bengali or bosnian or bulgarian or burmese or catalan or chinese or croatian or czech or danish or dutch or esperanto or estonian or finnish or flemish or french or gaelic, scots or georgian or german or greek or hausa or hebrew or hindi or hungarian or icelandic or indonesian or interlingua or italian or japanese or kirghiz or korean or latin or latvian or lithuanian or macedonian or malay or marathi or masai or multilingual or norwegian or persian or polish or portuguese or pushto or rumanian or russian or serbian or slovak or slovene or spanish or swahili or swedish or tagalog or tamil or telugu or thai or turkish or ukrainian or undetermined or urdu or vietnamese or welsh) (109)
**85**  84 and 83 (28)
**86**  83 or 85 (3842)
**87**  86 not 53 (2703)
