# Supplementary material for: How effective are remote and/or digital interventions as part of alcohol and drug treatment and recovery support? A systematic review and meta‐analysis
Source: Addiction. 2025 Mar 24;120(8):1531–50. doi: 10.1111/add.70021 (PMC12215248; doi:10.1111/add.70021)
Supplement: Supplementary file 4 — Appendix 4: Approaches to screening using priority screening and machine classifiers. [file ADD-120-1531-s005.docx]

**APPENDIX 4 Approaches to screening using priority screening and machine classifiers**

1. **Initial update search from original map (July 2021)**

The search was run in July 2021 and a machine learning classifier built within EPPI-Reviewer was used to filter the results. The classifier was trained on the screening decisions of the original map. Records with a relevancy ranked score of 10 or more with a publication date since 2018 were screened (the original map searches were undertaken in April 2019). Furthermore, unscreened records prior to 2018, and with a relevancy ranked score of 10 or more were added to the priority screening pool along with the expanded searches (in 2). Just 276 of these remain unscreened. All records were screened from forward and backward citation searching, related-item searches, handsearches and title-only records.

1. **Expansion searches (November 2021)**

The search results comprised three components, owing to the way the searches were operationalised. Priority screening was undertaken within EPPI-reviewer. This approach trains a machine classifier using a subset of known includes and excludes, and the classifier then lists the remaining references to be screened in order of their likely relevance. As reviewers screen the list of references the classifier incorporates the new screening decisions to re-rank the screening list and increase accuracy. Screening ceases when a given stopping criterion is reached. To implement this approach, a number of steps were taken prior to undertaking priority screening to inform a decision on stopping screening. First, random samples were screened and baseline inclusion rates were calculated for each of these components. Second, Lenth’s power calculator^^[[1]](#footnote-1)^^ was used to calculate the number of references required to be screened randomly in order to provide an estimate for an initial estimated inclusion rate. Third, once this quantity of references had been screened, the baseline inclusion rate was determined along with a further power calculation to check the significance of the sample in determining this rate. As this baseline inclusion rate was within the margin of error of the initial predicted inclusion rate, and a sufficient quantity of references had been screened, we took the baseline inclusion rate as a reasonable indicator of the likely number of eligible studies in the corpus of records. The following table summarises the baseline inclusion rates and actual inclusion rates prior to ceasing screening.

| **Search component** | **Sample size (n)** | **Baseline inclusion rate (%)** | **Margin of error for n @95% Confidence Interval (%)** | **Records available to screen** | **Includes from screening** | **Actual inclusion rate (%)** |
| --- | --- | --- | --- | --- | --- | --- |
| Part 1 | 442 | 3.8 | 1.6 | 2565 | 106 | 4.1 |
| Part 2 | 344 | 3.5 | 1.7 | 1374 | 38 | 2.7 |
| Part 3 | 325 | 2.8 | 1.7 | 6448 | 164 | 2.5 |

The decision to stop screening was based on two pieces of information. The first was the predicted number of eligible studies based on the baseline inclusion rate. Once that number of includable studies was met or exceeded, we could make the reasonable assumption that we had identified all (or the vast majority) of all relevant records. The second was the observation that no new records were being identified as screening down the list progressed (via a graphical display of the inclusion rate over time that was observable during screening).

Once screening ceased, a classifier was built based on the training parameters of the priority screening and this was applied to the unscreened references (n=6047), to observe the relevance-ranking of the unscreened references, and the eight records that were ranked above a score of 29 were scanned.

**3. July/August 2023 update screening**

**3.1 Database update search and citation and related item searches (July 2023)**

4,162 records were filtered to 2227 records after applying machine-learning classifiers. Of these, 1704 records were screened using priority screening. This process is described as follows:

1) A user-built classifier was trained on certain screening decision of records from the original systematic map (i.e. with a broader inclusion criteria than the map ), using EPPI-Reviewer. The user-built classifier was designed to exclude records that were not digital interventions on prevention, treatment, recovery from substance misuse.  The threshold relevance score of under 20 was determined as a conservative threshold and was informed by testing the classifier performance on the 49 includes from the original review (and which were not used in the training of the user-built classifier). The relevance scores for these were between 40-99.

2) The Original RCT classifier within EPPI-Reviewer was applied to a subsample of the records from database searches that had not already been indexed as 'randomised' in the databases (exclusion of records at score of below 10, which we understand is similar to the Cochrane validation at 98.5% recall). This approach was informed by testing on the 49 includes from the original review.  This step helped remove records that are unlikely to be RCTs from available references.

These two approaches yielded 2,227 records to screen. A pragmatic decision to cease screening was taken after observing a plateau in the prioritised screening rate. At this point, the overall include rate at title and abstract screening was noted as 57/1704 (3.3%).

There were three components of searches and the following table summarises the references retrieved, which classifiers were applied, and the remaining records that were added to the priority screening pool.

| **Search component** | **Records retrieved** | **Original RCT classifier apply and exclude score of <10** | **User-built Classifier** | **Records to add to priority screening pool:** |
| --- | --- | --- | --- | --- |
| Citation and related item searches on OpenAlex | 2394 | No | Yes | 1280 |
| Searches with “random*” or “clinical trial” (etc) in any searchable database field | 2867 refs, 1013 after de-duping | No | Yes | 507 |
| Update searches without “random*” or “clinical trial” (etc) in any searchable database field | 9664 refs, 3460 after de-duping | Yes | Yes | 445 |

**3.2 Recommender searches (OpenAlex).**

3238 records after duplicate removal were obtained from this method, of which 257 records were screened using priority screening.

This search comprised of OpenAlex Keep-Up-To-Date recommender searches in EPPI-Reviewer (12 July and 21 August 2023 updates). The 3238 records were prioritised from the recommender in two ways 1) user-built classifier developed in 3.1 (0.7 threshold) and 2) auto-classifier (0.97 threshold).  Once imported into EPPI-Reviewer the first 257 records of the 3238 records were screened using priority screening and no new relevant records were identified.  This search was undertaken to increase the currency of the update search.  It only drew on records added to OpenAlex between 4 June -20 August 2023 only, and therefore the number of records screened within this pool was low as it was not expected to identify many new records published within this short timeframe.  Ceasing screening was informed by the nature of the records presented to the screeners.

1. Russ Lenth’s power calculator [http://www.stat.uiowa.edu/~rlenth/Power/index.html](https://eur01.safelinks.protection.outlook.com/?url=http:%2F%2Fwww.stat.uiowa.edu%2F~rlenth%2FPower%2Findex.html&data=02%7C01%7Cc.stansfield%40ucl.ac.uk%7C9123bf9529a84d9fa40308d6d4900c28%7C1faf88fea9984c5b93c9210a11d9a5c2%7C0%7C0%7C636930112468917466&sdata=gAnKN%2BMziiKNphbtl9aoqO8ozFuEJlvTKipgTrldCcQ%3D&reserved=0) test “CI for one proportion” [↑](#footnote-ref-1)
