# Supplementary material for: How effective are remote and/or digital interventions as part of alcohol and drug treatment and recovery support? A systematic review and meta‐analysis
Source: Addiction. 2025 Mar 24;120(8):1531–50. doi: 10.1111/add.70021 (PMC12215248; doi:10.1111/add.70021)
Supplement: Supplementary file 5 — Appendix 5: Data extraction (coding) tool. [file ADD-120-1531-s006.docx]

**Appendix 5: Data extraction (coding) tool**

|  | **Item** | **Codes** |
| --- | --- | --- |
|  | Aim of the study | -Specify |
|  | Substance use disorder of population targeted | a)Alcohol  b)Drugs  -Cocaine  -Opioids  -Cannabis  -Methamphetamine  -Other / mixed drugs / unclear  c)Mixed drugs and alcohol |
|  | Population: Who are the study participants? | -Inclusion criteria/eligibility (specify) |
|  | Does the study focus on a specific population group? | - prison population  -pregnant women  -other specific population (specify) |
|  | Do the authors report the sex of participants? | - female only  -male only  -mixed-sex  -sex unclear / not reported |
|  | Do the authors report the ethnicity of participants? | -Ethnicity reported  -Ethnicity not reported / unclear |
|  | Do the authors report the age of participants? | - Age reported  -Age not reported / unclear |
|  | Do the authors report the socio-economic status of participants? | - SES reported  -SES not reported |
|  | Anything else notable that we should capture about population? | - Yes (note details)  -No |
|  | Broad intervention type | -Remote recovery support  -Self-guided therapy  -Remote talking therapy  -Other |
|  | Intervention: Which remote therapy/therapies is/are evaluated? | a)Is this a multi-intervention arm trial? (add v brief description)  -Yes, there are 3+ arms - and there are multiple remote therapy intervention arms  -Yes, but there is only one remote therapy intervention arm  -Only a single intervention and single comparator  b)Remote intervention arm 1  *Is arm 1 intervention in addition to standard care?*  -Yes- arm 1 intervention group receive standard care + remote therapy (standard care details)  -No - arm 1 intervention group receive remote therapy as a substitute for standard care  -No - there is no standard care in either arm 1 intervention or comparison  -other/not sure  - Arm 1 remote therapy intervention package content (details)  -Arm 1 remote therapy package duration/frequency/amount (details)  *Intervention length - Arm 1 (details)*  - >1 month but ≤ 3 months (e.g. 8 week intervention - add details)  - > 3 months but ≤ 6 months (e.g. 24 weeks - add details)  -> 6 months but ≤ 12 months (e.g. 8 months - add details)  -> 12 months (e.g. 15 month intervention - add details)  c)Remote intervention arm 2  -N/A only 1 remote intervention arm  -Arm 2 remote therapy intervention content (details)  -Arm 2 duration/frequency/amount of intervention (details)  *Is arm 2 in addition to standard care?*  -Yes arm 2 receive standard care + remote therapy  -No - arm 2 intervention group receive remote therapy as a substitute for standard care  -No - there is not standard care in either arm 2 or comparison  -Other / not sure  *Intervention length - Arm 2 (details)*  -≤ 1 month (e.g. 4 week intervention or less - add details)  ->1 month but ≤ 3 months (e.g. 8 week intervention - add details)  -> 3 months but ≤ 6 months (e.g. 24 weeks - add details)  -> 6 months but ≤ 12 months (e.g. 8 months - add details)  -> 12 months (e.g. 15 month intervention - add details)  d)Remote intervention arm 3  -Anything else we should capture about the intervention arms? |
|  | Intervention timing | - All digital intervention is only treatment received (i.e. not alongside or after residential or outpatient) (n=6)  -All alongside inpatient / outpatient treatment (n=25)  -All intervention as aftercare / post inpatient or outpatient treatment (n=21) |
| 1. 11. | What is the control arm (for purposes of this review)? | a)Are there multiple comparison arms? (i.e. non-remote therapy arms)  -No - only a single comparator  -Yes - several non-remote therapy comparators (details)  b) Content of control arm(s)  -Control group get standard care only  - Control group get alternative intervention (details)  -Anything else we should capture about the control arm(s)? |
|  | Comparators | -#1 Superiority: Remote aftercare vs no aftercare  -#2 Non-inferiority: Remote aftercare / treatment vs f2f aftercare / treatment  -#3 Superiority: Remote is 'Add-on' to standard treatment: Remote treatment + F2F treatment vs F2F treatment only  -#4 Non-inferiority: Remote partially replaces f2f: Remote therapy partially replaces f2f treatment vs fully f2f treatment  #5 Superiority: Equal time sham / placebo control: Remote therapy / aftercare vs equal time sham / placebo |
|  | comparison types | -When RT is added to f2f treatment does it reduce the likelihood of relapse? (n=11 studies)  - When RT replaces or partially replaces f2f treatment does it reduce the likelihood of relapse? (n=4 studies)  - When RT is added to f2f treatment does it reduce the amount of drugs / alcohol people use? (n=14 studies)  -When RT replaces or partially replaces f2f treatment does it reduce the amount of drugs / alcohol people use? (n=9 studies) |
|  | Outcomes (types) | -% TIME abstinent  - Odds of relapsing |
| 1. 12. | Outcomes (Measurement details) | a)Substance use outcomes measured (specify)  -Self report only  -Toxicology only (e.g. urine / breath / blood)  -Self report & toxicology  -Other substance use measure  b)How are substance use outcomes measured? (tick all that apply)  -Dichotomous measure of abstinence- e.g. % of participants abstinent or not (% of participants who remained abstinent)  -Continuous - longest period of continuous abstinence (e.g. total consecutive weeks of abstinence)  -Any other continuous measure of abstinence e.g. how many days / weeks abstinent - number, % or rate of days / weeks abstinent in a given time period  - Other non-abstinence outcomes - e.g. number of drinks per day  -Anything else we should capture about the outcomes?  c) Outcome measure time points  *When are outcomes measured in relation to the intervention? (tick ONE only)*  -during and / or at the end of the intervention only  -Includes follow up measurements after the intervention has ended  *At which point(s) were outcomes measured post-baseline? (tick all that apply)*  -≤ 3 months post baseline  ->3 months but ≤ 6 months post baseline  - > 6 but ≤12 months post baseline  ->12 months but ≤ 18 months post baseline  -18+ months post baseline  -other (specify) |
|  | Timepoint | -End of intervention  - Follow-up after end of intervention |
|  | Length of outcome measurement period | - 1 week  - 2 weeks  - 4 weeks  - 8 weeks  - 12 weeks  - 16 weeks  - 6 months  - 12 months |
|  | Reporting of outcome in paper | -Reported as relapse in the paper  -Reported as abstinence in the paper |
