# Supplementary material for: How effective are remote and/or digital interventions as part of alcohol and drug treatment and recovery support? A systematic review and meta‐analysis
Source: Addiction. 2025 Mar 24;120(8):1531–50. doi: 10.1111/add.70021 (PMC12215248; doi:10.1111/add.70021)
Supplement: Supplementary file 6 — Appendix 6: Risk‐of‐Bias 2 assessments according to outcomes (n=34 RCTs, 42 interventions). [file ADD-120-1531-s002.docx]

**APPENDIX 6 Risk-of-Bias 2 assessments according to outcomes (n=34 RCTs, 42 interventions)**

| **+** | Low RoB | **?** | Some concerns | ─ | High RoB |
| --- | --- | --- | --- | --- | --- |

|  | **Study** | **Outcomes** | **Domain 1.**  **Risk of bias arising from the randomisation process** | **Domain 2.**  **Risk of bias due to deviations from the intended interventions** | **Domain 3. Missing outcome data** | **Domain 4.**  **Risk of bias in measurement of the outcome** | **Domain 5.**  **Risk of bias in selection of the reported results** | **Overall risk of bias** |
| --- | --- | --- | --- | --- | --- | --- | --- | --- |
|  | **Brooks (2010)** | Days of use | **?** | **─** | **+** | **─** | **?** | **─** |
|  | **Campbell (2014)** | Relapse | **+** | **+** | **+** | **+** | **+** | **+** |
|  | **Campbell (2014)** | Days of use | **+** | **+** | **+** | **+** | **+** | **+** |
|  | **Carroll (2008)** | Days of use | **?** | **?** | **─** | **─** | **?** | **─** |
|  | **Carroll (2014)** | Relapse | **?** | **?** | **+** | **?** | **?** | **?** |
|  | **Carroll (2014)** | Days of use | **?** | **?** | **+** | **+** | **?** | **?** |
|  | **Chaple (2016)** | Days of use | **?** | **─** | **─** | **─** | **?** | **─** |
|  | **Christensen (2014)** | Days of use | **?** | **+** | **+** | **+** | **?** | **?** |
|  | **DeMartini (2018)** | Relapse | **+** | **+** | **+** | **+** | **?** | **?** |
|  | **Fals-Stewart (2010)** | Days of use | **?** | **+** | **─** | **─** | **?** | **─** |
|  | **Farabee (2013)** | Relapse | **?** | **+** | **─** | **+** | **?** | **─** |
|  | **Farabee (2013)** | Days of use | **?** | **+** | **─** | **─** | **?** | **─** |
|  | **Farren (2014)** | Days of use | **+** | **─** | **─** | **─** | **?** | **─** |
|  | **Farren (2021)** | Days of use | **?** | **+** | **─** | **?** | **+** | **─** |
|  | **Godley (2010)** | Days of use | **?** | **?** | **─** | **─** | **?** | **─** |
|  | **Gonzales (2014)** | Relapse | **?** | **─** | **─** | **─** | **?** | **─** |
|  | **Graser (2021)*** | Relapse | **?** | **+** | **+** | **─** | **?** | **─** |
|  | **Gustafson (2014)** | Relapse | **+** | **+** | **─** | **─** | **?** | **─** |
|  | **Hyland (2023)** | Days of use | **+** | **+** | **─** | **?** | **─** | **─** |
|  | **Johansson (2021)** | Days of use | **+** | **+** | **+** | **+** | **─** | **─** |
|  | **Kelpin (2022)** | Relapse | **?** | **+** | **+** | **?** | **+** | **?** |
|  | **Kelpin (2022)** | Days of use | **?** | **+** | **+** | **?** | **+** | **?** |
|  | **Kiluk (2016)†** | Days of use | **+** | **?** | **+** | **?** | **?** | **?** |
|  | **Kiluk (2018a)** | Relapse | **?** | **?** | **─** | **+** | **?** | **─** |
|  | **Kiluk (2018a)** | Days of use | **?** | **?** | **─** | **─** | **?** | **─** |
|  | **Lucht (2020)** | Relapse | **+** | **+** | **+** | **?** | **?** | **?** |
|  | **Lucht (2020)** | Days of use | **+** | **+** | **+** | **?** | **?** | **?** |
|  | **McKay (2004)** | Relapse | **?** | **?** | **+** | **─** | **?** | **─** |
|  | **McKay (2004)** | Days of use | **?** | **?** | **+** | **─** | **?** | **─** |
|  | **McKay (2010)†** | Relapse | **+** | **?** | **+** | **?** | **?** | **?** |
|  | **McKay (2013a)†** | Relapse | **+** | **+** | **+** | **─** | **?** | **─** |
|  | **McKay (2022)*** | Relapse | **+** | **─** | **─** | **─** | **─** | **─** |
|  | **Mundt (2006)†** | Relapse | **?** | **─** | **─** | **─** | **?** | **─** |
|  | **Paris (2018)** | Days of use | **+** | **+** | **+** | **+** | **?** | **?** |
|  | **Taştekin (2022)** | Relapse | **?** | **─** | **─** | **─** | **?** | **─** |
|  | **Tetrault (2020)** | Days of use | **?** | **+** | **─** | **─** | **?** | **─** |
|  | **Tiburcio (2018)** | Days of use | **+** | **?** | **─** | **─** | **?** | **─** |
|  | **Timko (2019a)** | Days of use | **?** | **+** | **─** | **─** | **?** | **─** |
|  | **Timko (2019b)** | Days of use | **?** | **+** | **─** | **─** | **?** | **─** |
|  | **Verduin (2013)** | Relapse | **?** | **+** | **─** | **─** | **?** | **─** |
|  | **Wolitzky-Taylor (2018)** | Days of use | **?** | **+** | **─** | **─** | **─** | **─** |

*****RCT with 3 intervention arms; **†**RCTs with 2 intervention arms
